# Fractionation of Technical Lignin from Enzymatically Treated Steam-Exploded Poplar Using Ethanol and Formic Acid

**DOI:** 10.1021/acsapm.2c01665

**Published:** 2022-11-24

**Authors:** Riku Maltari, Jussi Kontro, Klaus Koivu, Muhammad Farooq, Joona Mikkilä, Rui Zhang, Kristiina Hildén, Jussi Sipilä, Paula A. Nousiainen

**Affiliations:** † Department of Chemistry, University of Helsinki, P.O. Box 55, A. I. Virtasen Aukio 1, Helsinki FI-00014, Finland; ‡ Department of Microbiology, University of Helsinki, P.O. Box 56, Viikinkaari 9, Helsinki FI-00014, Finland; § Department of Bioproducts and Biosystems, Aalto University, Vuorimiehentie 1, Espoo FI-02150, Finland

**Keywords:** biorefinery lignin, solvent fractionation, NMR structural analysis, thermal properties, elemental analysis

## Abstract

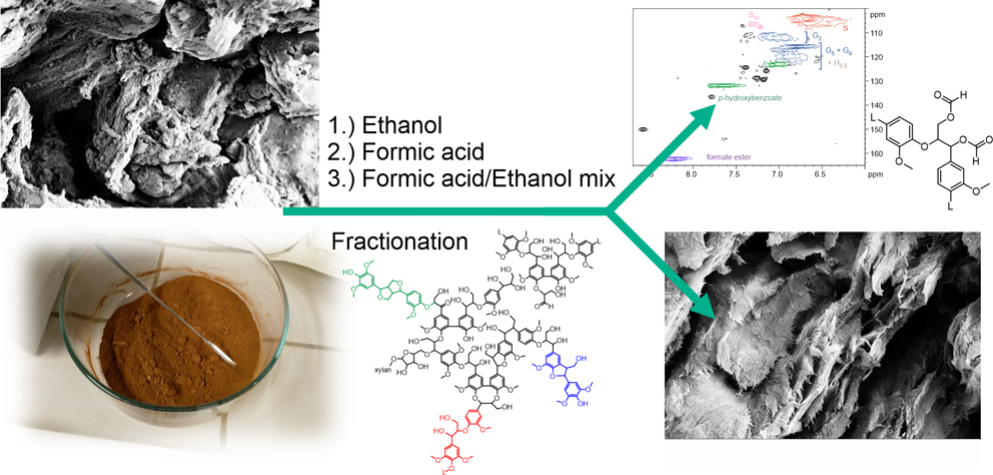

Lignocellulosic biorefineries produce lignin-rich side streams with high valorization potential concealed behind their recalcitrant structure. Valorization of these residues to chemicals, materials, and fuels increases the profitability of biorefineries. Fractionation is required to reduce the lignins’ structural heterogeneity for further processing. We fractionated the technical biorefinery lignin received after steam explosion and saccharification processes. More homogeneous lignin fractions were produced with high β-O-4′ and aromatic content without residual carbohydrates. Non-toxic biodegradable organic solvents like ethanol and formic acid were used for fractionation and can be adapted to the existing biorefinery processes. Macromolecular properties of the isolated fractions were carefully characterized by structural, chemical, and thermal methods. The ethanol organosolv treatment produced highly soluble lignin with a reasonable yield, providing a uniform material for lignin applications. The organosolv fractionation with formic acid and combined ethanol-formic acid produced modified lignins that, based on thermal analysis, are promising as thermoresponsive materials.

## 1 Introduction

The importance of converting current fossil-based economy to biobased circular economy has been globally recognized, supported, and adopted into international and national public policies.^1^ More sustainable industrial processes need to be developed to ensure the availability of fuels and platform chemicals in the future. Current production of commodities is largely based on non-renewable raw materials, such as fossil resources, which are estimated to be depleted or their availability and price becomes less competitive.^2^ Another important constraint on the use of fossil raw materials is the growing concern about greenhouse emissions that has shifted the focus to renewable feedstocks.^2^ The chemical, material, and fuel industries have increased the use of sustainable lignocellulosic biomass-based carbon because lignocellulose is the most abundant renewable source for organic raw materials globally.^3^

The major compounds in cell walls of lignified plant tissues are cellulose, hemicellulose, and lignin. Together, they form a complex lignocellulose matrix, which provides structural rigidity for wood. The major component of lignocellulose is cellulose, comprising ca. 40–50% of the dry weight of wood.^4^ Currently, cellulose is the most widely utilized part of wood, while fewer applications have been developed for more complex lignin and hemicelluloses. In pulp and paper industries, cellulose is separated from lignocellulose through the Kraft process, which forms a huge lignin side stream fraction with the condensed structure and relatively high sulfur content that limit its valorization prospects.^5^ Therefore, non-sulfur lignins obtained from cellulosic biorefineries are more suitable for catalytic valorization.^6^

The second generation (2G) ethanol biorefineries are based on a variety of raw materials including wood-based biomass and agricultural wastes^7^ that are produced in large quantities worldwide and can have negative environmental impacts if discarded.^8^ Pretreatment of lignocellulosic biomass is an important step in biorefineries, and physicochemical methods such as steam explosion (SE) and acidic extrusion have been used as good alternatives for chemical pulping of lignocellulosic biomass. SE produces more easily valorizable sulfur-free lignin fractions as the side product.^9,10^ SE lignins also have generally less structural alterations than ionic liquid and deep eutectic solvent lignins.^11,12^ In these processes, the solvent recovery can be technically and economically challenging.^11^ However, the production yields in SE generally remain low and the side streams should be more efficiently used and valorized to obtain value-added chemicals and materials to increase the profitability of biorefineries.

Technical lignins are heterogenic in structure, and they differ in their purity and chemical properties. Therefore, additional purification steps are needed to narrow their chemical properties. In general, three different types of purification methods have been used: selective precipitation, organic solvent dissolution, and membrane fractionation. Solvent fractionation has recently regained interest in efforts to produce more homogenous lignin fractions.^13−17^ The insolubility of large part of the lignin-rich material is considered an obstacle to the lignin modification for different industrial applications.^12^ Insolubility is likely caused either by condensation or by physically or covalently attached carbohydrates in the lignin polymer.^18^ Several studies have been published on the solubility of technical lignins in different organic solvents and solvent combinations with respect to their solubility parameters.^19−21^ Notable examples of solvent fractionation techniques for technical lignins are sequential-fractionation-based methods, such as the use of ethyl acetate, ethanol, methanol, and acetone,^22^ acetic acid and water in different ratios,^14,17^ and dichloromethane, n-propanol, methanol, and methanol-dichloromethane mixture.^13^ Solvent mixtures like acetone-methanol and tetrahydrofuran (THF)-methanol have also been used.^16^ Almost without exception, lignin is recovered in these experiments by precipitation with an appropriate antisolvent like water or hexane.^16,22^

Fractionated lignin with low molecular weight (MW) and high aliphatic hydroxyl-group content can be chemically or enzymatically modified to produce higher value materials.^23−27^ The main products expected from fractionated lignin are adhesives, phenolic resins, bio-oils, fuel additives, and platform chemicals.^6,7,12^ Moreover, the soluble lignin fractions are needed for producing colloidal lignin nanoparticles for biomaterials in various downstream applications, such as carriers of bioactive molecules, layer-by layer assemblies, or thermoset resins and composites.^28^ Meanwhile, fractionated lignin with a high MW and uniform structure has been proposed to be a good starting material for carbon fiber production.^14^

In this study, we focus on residual hardwood biorefinery lignin produced from the SE process in a 2G bioethanol plant. We explored three different-polarity organic-solvent extraction methods that are non-toxic using hot ethanol, formic acid, or their 1:1 mixture to fractionate lignin to low and high MW fractions. To our knowledge, systematic studies have not been performed on such solvent systems for biorefinery SE lignins. These methods were compared to a lignin purification method with aqueous NaOH.^12^ We show that the solvent extractions resulted in high-quality lignin fractions with reasonably high yields. The fractions were structurally characterized by gel permeation chromatography (GPC), 1D and 2D nuclear magnetic resonance spectroscopy (NMR), ^31^P NMR, infrared spectroscopy (IR), pyrolysis-GC-MS (Py-GC-MS), elemental analysis, thermogravimetry (TGA), and differential scanning calorimetry (DSC). Ethanol-treated fractions showed superior properties with narrow molecular weight distribution (MWD) and high content of β-O-4, β-5, and β–β lignin interunit bonds, whereas formic acid extractions produced higher lignin yields with altered lignin structures.

## 2 Materials and Methods

### 2.1 Raw Materials and Chemicals

The wet SE lignin-rich residue was received from Italian Bio Products SRL (Crescentino, Piedmont, Italy), where it was produced from poplar feedstock as a side product in the 2G bioethanol process using PROESA technology. According to the manufacturer’s report, the crude lignin moisture content was 67%, and it was characterized on a dry basis in terms of residual sugars (30 wt %), Klason lignin (55 wt %), ashes (2 wt %), and other components (13 wt %). In the residual sugars, 95% were hexoses (mainly glucan from cellulose) and 5% pentoses (mainly xylan from hemicelluloses). The wet lignin cake was washed with distilled water at 80–90 °C for 1 h, filtered, and dried in ambient temperature and pressure over the course of 1 week, and later in the text, it is referred to as SEL (SE lignin). The water content of SEL (2% w/w) was determined by lyophilization. All used chemicals, which are not explicitly described in the materials and methods, are listed with purities and suppliers in the Supporting Information.

### 2.2 Extraction Procedures

The extractions of SEL were performed at refluxing temperature with the following organic solvents: 1) ethanol (VWR, 99%), 2) formic acid (Aldrich, 99%), or 3) a mixture of formic acid and ethanol (1:1 v/v). For ethanol and ethanol-formic acid extractions, approximately 2 g of SEL was refluxed for 4 h with a lignin-solvent ratio of 1 g/10 mL. Formic acid extraction was performed with a similar protocol except for the reflux time of 30 min instead of 4 h. The solids were filtered out from the extraction mixture before cooling, followed by evaporation of solvents. The extracted fractions were dried to a constant weight under vacuum using an oil pump. The yields of the fractions were calculated without considering the water content of SEL (2%). The base-treated lignin was prepared using a modified protocol from Hyväkkö et al.;^29^ see details in the Supporting Information. All extractions were performed in duplicate. The fractions were named systematically based on the lignin (**I**BP), extraction solvent [**E**thanol, **F**ormic acid, **M**ixture (1:1) of both and **B**ase-extracted], and MW based on solubility in the extraction solvent (**L**ow for soluble fraction and **H**igh for insoluble fraction).

Extractive contents for IEL and SEL were determined after lyophilization followed by Soxhlet extraction overnight with *n*-hexane (Honeywell, Riedel-de-Haën, HPLC, 97%). The extraction yields were 2% and 1% w/w, respectively.

### 2.3 Gel Permeation Chromatography

GPC experiments were performed according to Kontro et al.,^30^ and details are given in the Supporting Information.

### 2.4 Infrared Spectroscopy

Infrared spectra (400–4000 cm^–1^) were recorded on a Bruker Alpha FT-IR spectrometer (Bruker Optics) with a platinum single reflection diamond attenuated total reflection (ATR) accessory. Altogether, 24 scans per sample were collected with a resolution of 4 cm^–1^.

### 2.5 1D and 2D Nuclear Magnetic Resonance Spectroscopy

The acetylated samples were dissolved in DMSO-*d*_6_ and measured using a Bruker AVANCE III 500 MHz NMR spectrometer with a BBFO broad band probe. All acetylated samples were fully soluble in DMSO-*d*_6_. The residual solvent peak (δ_C_ 39.52 and δ_H_ 2.50) was used as an internal reference. All experimental details are given in the Supporting Information [^1^H, ^13^C, heteronuclear single quantum correlation (HSQC), heteronuclear single quantum correlation–total correlation spectroscopy (HSQC-TOCSY), heteronuclear multiple bond correlation (HMBC), and ^31^P].

### 2.6 Elemental Analysis

Measurements were performed on an Elementar Analysensysteme (HANAU) model vario MICRO cube, operated in CHNS mode. Sulfanilamide was used as a standard, and nitrogen, carbon, hydrogen, and sulfur were detected as N_2_, CO_2_, H_2_O, and SO_2_ gases with a thermal conductivity detector and He as a carrier gas. The samples’ oxygen content was calculated by difference between the sample weight and the C, H, N, and S content.

### 2.7 Thermogravimetry

Thermogravimetric analysis (TGA) was used to study thermal degradation curves of SEL and its fractions. A Mettler Toledo TGA/SDTA851e/SF/1100 with Julabo cooler model ED was used. The experimental protocol is given in the Supporting Information.

### 2.8 Differential Scanning Calorimetry

Glass transition temperatures (*T*_g_) of samples were determined using a TA Instruments Q200 DSC coupled to a RCS90 cooling system. The experimental protocol is given in the Supporting Information.

### 2.9 Pyrolysis Gas Chromatography–Mass Spectrometry

Py-GCMS experiments were performed according to Kontro et al.,^31^ and the details are given in the Supporting Information.

### 2.10 Lignin Content by Acetyl Bromide Assay

Acetyl bromide (AcBr) assays were performed according to Hyväkkö et al.^29^ A Varian Cary 50 Conc UV-VIS spectrometer at 280 nm was used for all measurements.^41^ The detailed protocol is given in the Supporting Information.

### 2.11 Ash Content Analysis

Ash contents in lignin were determined by accurately weighing 10–20 mg of desiccator-dried lignin to pre-weighed Al_2_O_3_ TGA vessels. The vessels were placed in a Netzsch STA 449F3 TGA/DSC. The TGA was heated to 1000 °C at the rate of 40 °C/min using an air atmosphere. The ash content was gained by measuring the residual mass.

### 2.12 Field Emission Scanning Electron Microscopy

The original SEL and all fractionated lignin samples’ surface and cross-sectional morphology were investigated with a Zeiss Sigma VP with a Schottky Field Emission Gun (FEG) scanning electron microscope (Zeiss Sigma, Germany). The detailed protocol is given in the Supporting Information.

## 3 Results and Discussion

### 3.1 Material Observations

The aim of this work was to produce high-quality lignin for further valorization of biorefinery lignin side stream to fuels or aromatic platform chemicals.^30−32^ Solvent fractionation methods allow for solvent recycling in closed circulation systems through distillation, while simultaneously, the lignin fraction is isolated as a part of the process. To evaluate the success of the treatments, the following quality criteria were defined for the isolated lignin: (1) Low residual carbohydrate content, which is generally expected to reduce industrial applicability of lignin.^16^ (2) Intact lignin phenylpropane side-chain structure, since the aliphatic hydroxyl functional groups can be further modified.^6,26^ (3) High solubility, which translates to increased reactivity. (4) High yield to increase the value and profitability of the process. In this study, three inexpensive non-toxic organic solvent systems were applied in fractionation. Previously, ethanol extraction has been used on SE lignin without heating.^33^ In our experiments, we detected a clear increase in lignin solubility in ethanol refluxing conditions that assist dissolution by increased intermolecular interactions.^34^ In comparison, formic acid has been studied as a method of delignification or biomass fractionation,^15,35,36^ but not lignin fractionation.

In SE conditions at high temperature, acidolytic reactions give rise to potential degradation of lignin structures and condensation reactions producing a complex reaction pattern.^37^ Furthermore, the crude lignins (SEL) Klason lignin content was 55%, and it contained a significant amount of insoluble carbohydrates (30%) due to an insufficient saccharification step. The FESEM morphological analysis of each of the isolated fractions evidenced that the isolated insoluble fractions contained fibrous materials. IBH especially showed bundles of fibers, and in IFH, these bundles were found partially separated (Figure S8). These insoluble fractions could be further circulated into a saccharification process after fractionation to increase the effectivity of the process. The mass yields of soluble fractions are given in Table 1 and expressed as both measured mass yields and wt % of the crude lignin’s Klason lignin content.

**Table 1:** Fraction Yields, Lignin Content, Ash Content, Elementary Composition, and MW Distribution Values of Soluble Lignin Fractions

					elemental composition wt %^a^						MWD		
fraction	yield %	yield % vs Klason	lignin % AcBr	ash %	C	H	N	O	H/C	O/C	*M*_n_	*M*_w_	PDI
SEL			55	2.1	54.5	6.0	0.9	38.5	1.32	0.94			
IEL	18.3 ± 0.6	33	95	0.6	62.8	6.2	0.3^a^	30.3	1.18	0.64	1080	2200	2.04
IFL	65.6 ± 0.6	120	108^b^	2.7	58.3	5.4	1.0	35.1	1.11	0.80	1700	7090	4.17
IML	42.9 ± 0.9	78	105^b^	1.9	56.8	5.6	0.9	36.6	1.18	0.85	1560	7290	4.67
IBL	35.3 ± 0.7	64	90	0.4	61.2	5.9	0.9	31.7	1.15	0.69	1390	5070	3.65

a S wt % below calibration range and O wt % calculated as the residue of CHNS.

b Lignin derivatization by formate esters disturbs the colorimetric analysis.

The soluble fraction yields from high to low are IFL > IML > IBL > IEL. In general, the high solubility indicates a lower MW and a lower degree of condensation in the lignin,^38^ but with these solvent systems, yields are inversely correlated with their solubility into polar organic solvents, such as acetone and dioxane. With formic acid, we reached up to 65% soluble material yield, which markedly exceeded Klason lignin content. This means that the IFL contains a substantial amount of impurities, such as carbohydrates, that can be hydrolyzed and solubilized under the acidic conditions caused by formic acid. Previously, it has been demonstrated that formic acid is an excellent solvent for lignin.^29,39^ The high solubility of lignin into formic acid is due to the formation of formate esters in most hydroxyl groups of lignin.^36,39^

In contrast to formic acid treatment, no acid catalysts or aqueous components were used in the hot ethanol solvent extraction, resulting in lignin solubilization without derivatization. Solubility in organic solvents has been shown to be dependent on the solvent’s polarity and hydrogen bonding capacity. Intermediate polarity solvents are more efficient in dissolving lignin compared to polar and non-polar solvents.^20^ In this regard, heating generally improves solubility of phenolic compounds unless the high temperature causes degradation of the molecular structure.^34^

Formic acid and ethanol treatments in lignin fractionation were compared to a simple alkaline isolation procedure using mild NaOH. In such mild alkaline conditions, the lignin structure is well preserved compared to acidic treatments, and the solubility of cellulose and hemicelluloses is very low.^29,40^ Furthermore, in a more concentrated alkaline solution and at elevated temperature, *p*-hydroxybenzoate esters in SEL were also cleaved and *p*-hydroxybenzoic acid was isolated from the lignin matrix by extraction (Supporting Information Figure S2). This enables the isolation of *p*-hydroxybenzoic acid as a platform chemical from poplar or aspen biorefinery side streams.

The carbon content of poplar wood has been approximated to vary between 48–52% because of the high carbohydrate content from cellulose and hemicellulose.^41^ Increased carbon content in a fractionation process typically indicates an increase in the lignin content and especially in the highest heating value.^42^ All soluble fractions had an increased carbon content (57–63%) compared to the initial SEL (54.5%) (Table 1). IEL had the highest carbon and hydrogen content, 62.8 and 6.2%, respectively. In line with our results, in elemental analysis, the carbon content of technical lignin has frequently been reported to be between 58–63% by mass depending on the origin of the sample.^43^ Reduction of oxygen content can be caused by lower carbohydrate content or chemical elimination of aliphatic hydroxyls. In this study, the formic acid fractions have the aliphatic hydroxyls modified as formate esters, which can be detected in the HSQC-NMR spectrum of IFL as discussed here in the section on structural analysis. The IEL and IBL fractions can be directly compared with SEL because these fractions do not have chemical modifications like IFL and IML, which have been derivatized by formic acid.

### 3.2 MW Analysis

In the analysis of biopolymers, GPC is an important tool to reveal the success of the separation methods and to determine the MWD of the isolated material.^44^ MWDs of solubilized fractions were determined (Table 1 and Figure S1), but the carbohydrate-containing insoluble residues were not analyzed. All samples were acetylated before measurement to improve solubility in THF eluent, altering the results for all fractions, and contributing to the imprecision of the method. All measured MW values are relative to the used standards, not absolute values. The IEL fraction had lower *M*_n_ (1080 Da) and *M*_w_ (2200 Da) than IBL, and the polydispersity as low as 2, indicating that this fraction had the highest uniformity. In the IEL fraction, the sample was fully soluble and the highest MW polymers were approximately 10 kDa.

In contrast, formic acid solubilizes most of the lignin and can, to some extent, also solubilize carbohydrates through acid hydrolysis, and as a result, the IFL fraction had the highest *M*_n_. The acidic conditions may also cause hydrolytic reactions that form condensed structures in lignin possibly increasing the MW.^36^ Both IFL and IML fractions had a distinct “tail” at 30–150 kDa area, also commonly found in Kraft lignins.^45^ One explanation for this could be the presence of residual formate esters in acetylated GPC samples, which results in incomplete dissolution in THF and the formation of aggregates. A small amount of insoluble material in the IFL and IML fractions was also removed by filtration before GPC measurement, likely shifting the result toward lower MW, because high MW lignin polymers tend to be less soluble in organic solvents.^19^ All isolated fractions had slightly lower *M*_w_ and *M*_n_ than the values for poplar milled wood lignin (*M*_n_ 4176 Da and *M*_w_ 13,250 Da) in literature.^46^ However, it has been broadly recognized that GPC is a relative method, and MWD measurements performed in different laboratories have large discrepancies, particularly when measuring SE lignin.^47^

### 3.3 Structural Analysis

**Figure 1:**
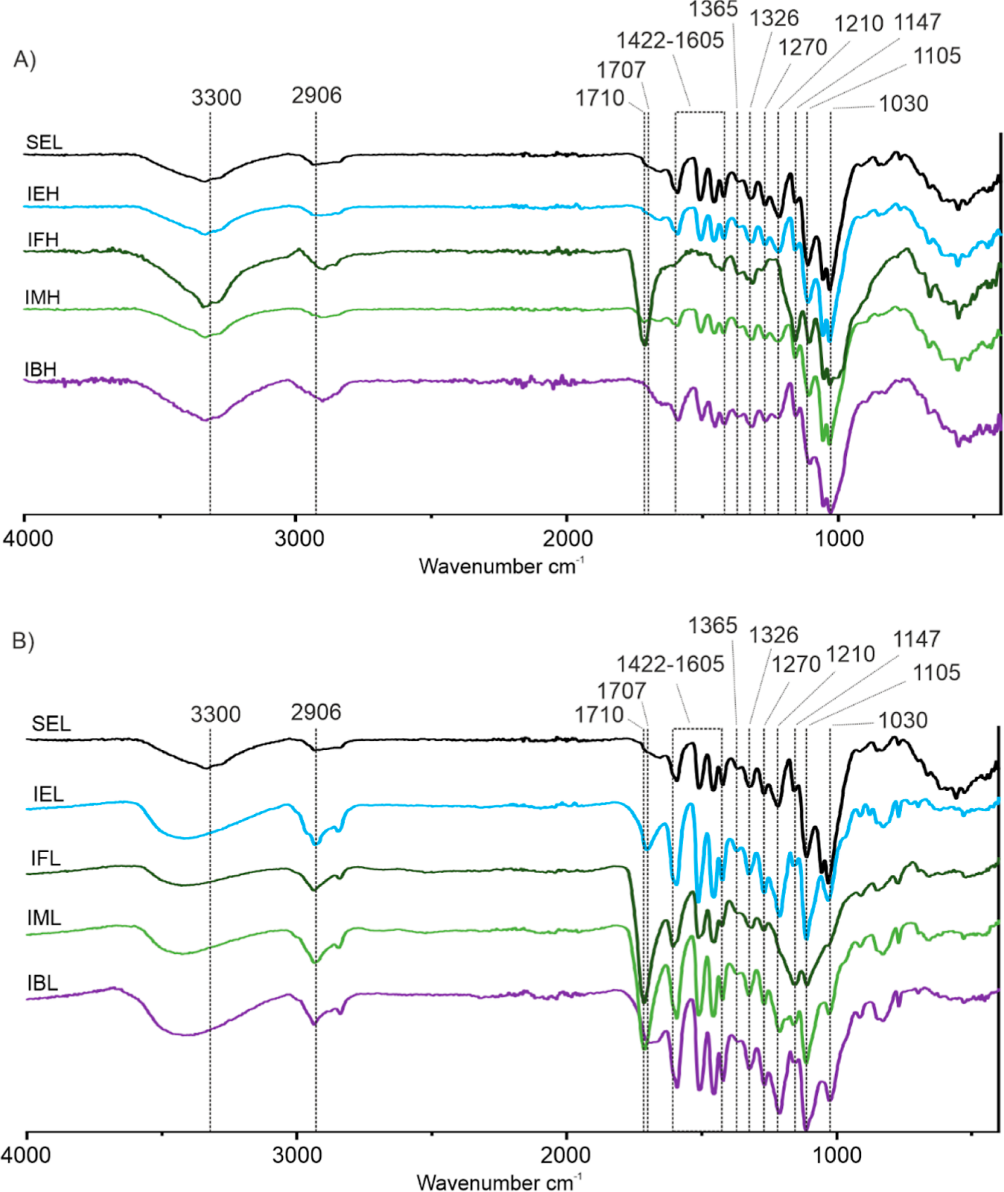
FTIR spectra of (A) insoluble fractions and (B) soluble fractions. Soluble fractions show more pronounced lignin bands, while insoluble fractions have diminished lignin bands and more intense carbohydrate bands.

In addition to the lignin yield, the molecular properties are essential parameters when evaluating different fractionation methods. The functional groups and chemical bonds present in the lignin polymer can be revealed by FT-IR analyses. The IR spectra of the starting material (SEL) and all fractions are shown in Figure 1A,B. In SEL and all high MW fractions, a strong band was detected at 1030 cm^–1^, which likely originates from hemicellulose or cellulose primary alcohol C–O deformation (Figure 1A).^48^ These peaks were notably reduced in all low MW fractions, suggesting primarily the presence of aromatic C–H bending bands at 1033 cm^–1^ and lignin side-chain γ-hydroxyl C–O bending, characteristic for lignin.^48^

In the fingerprint region, the signals were partially overlapping and therefore cannot be precisely identified. All solubilized lignin fractions had distinctly strong aromatic ring C–H and C=C stretching absorption bands in the spectral region of 1422–1605 cm^–1^ (1593, 1514, and 1422 cm^–1^) (Figure 1B).^49^ The aromatic C–H in-plane deformation band was at 1147 cm^–1^.^49^ In addition, the spectra contained absorption bands at 1460 and 1365 cm^–1^ originating from methoxyl C–H asymmetric and symmetric bending, respectively.^50^ Characteristic C–O bands were also found at 1326 cm^–1^ (stretching of 4-O in S or 5-substituted G)^50^ and 1270 (3-O stretching in G).^49^ The 1210 cm^–1^ band can be attributed to either C–O stretching or O–H plane deformation.^49,50^ Commonly identified C–H stretching bands can be found in all spectra at around 2906 cm^–1^,^51^ but with higher intensities in the soluble fractions, suggesting higher concentrations of aromatic methoxyls. The peak of the O–H stretching band at around 3300 cm^–1^ in SEL shifts to higher wavenumber 3400 cm^–1^ in the soluble fractions, indicating a change in the surroundings of the hydroxyl groups.^49−51^ In the soluble fractions IFL and IML (Figure 1B) and insoluble fractions IFH and IMH (Figure 1A), distinct unconjugated carbonyl C=O stretching signals were also detected at 1710 cm^–1^,^50^ likely because of formate esters. In IEL and IBL samples, the carbonyl band at 1707 cm^–1^ was found only as a slight shoulder representing most probably the *p*-hydroxybenzoate ester moiety found in the poplar lignin.

Based on the IR, it seems that the soluble fractions mainly contain lignin because the aromatic ring bands were more intense than the carbohydrate bands. In the insoluble fractions, some aromatic bands can still be found in other samples but IFH, which had almost none. This is in agreement with the estimation from mass balance that most lignin was solubilized in formic acid. Also, the presence of a large primary alcohol peak at 1030 cm^–1^ in IFH supports the idea that the insoluble residue is mostly carbohydrates.

**Figure 2:**
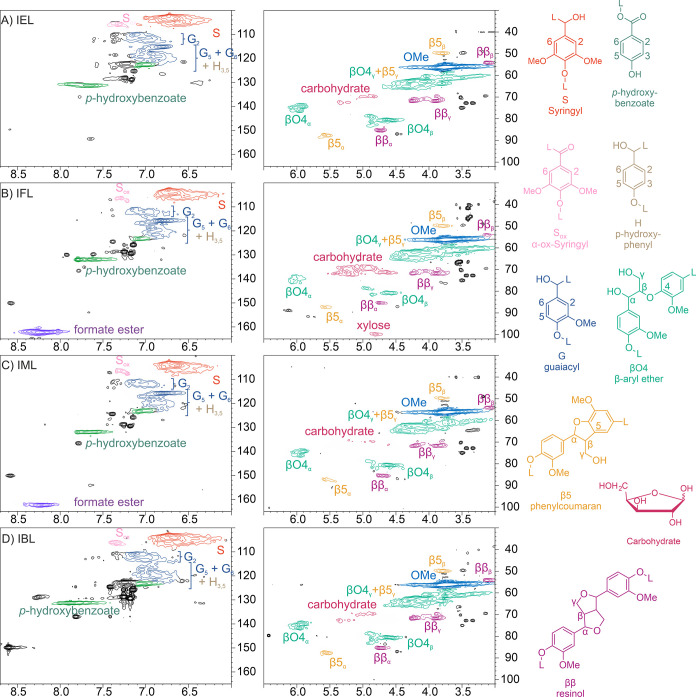
HSQC-NMR spectra of soluble fractions. (A) IEL, (B) IFL, (C) IML, and (D) IBL. Aromatic region (left) and side-chain region (right). All typical lignin signals are present in the soluble fractions. IFL and IML showed characteristic formate ester signals, indicating that they are not fully cleaved during acetylation. IFL has slightly pronounced carbohydrate signals and diminished β-O-4 α-signal, showing that formic acid has also solubilized carbohydrates.

Structural analysis by NMR spectroscopy allows for identification of lignin interunit linkages and aromatic unit composition as well as hydroxyl group quantification with ^31^P NMR. The HSQC-NMR analyses indicated clear differences in the structure of the extracted lignin fractions (Figure 2). Common features in the HSQC-NMR spectra include all typical lignin interunit bonds: arylglycerol β-aryl ether (β-O-4), resinol (β–β), and phenylcoumaran (β-5). The hardwood aromatic signals, that is, the syringyl and the guaiacyl groups, were well represented in all samples, including a small amount of α-oxidized aromatic units (Figure 2). The *para*-benzoate functional groups at the side-chain γ-position, which is typical in poplar and aspen lignins as well as some willow species, were also found in all spectra.^52,53^ These signals were also found in IBL (Figure 2D), indicating mild extraction conditions because, in general, the ester bonds can be cleaved with basic treatments.^54^

The HSQC-NMR spectrum of IEL-fraction showed high amounts of all typical lignin interunit bonds β-O-4, β–β, and β-5 in the ratio of 100:35:26, and it was almost free of carbohydrates (Figure 2A).^41^ The syringyl and guaiacyl group ratio was 100:96 according to integration of the aromatic region. Furthermore, some oxidized syringyl signals S2/6_ox_ (7%) were detected at δH/δC 7.4/106.0 ppm.^55^ Only the IFL fraction contained a notable amount of carbohydrates, indicated by their C–H correlation signals at δH/δC 4.5–5.5/67–73 ppm and the anomeric C–H signal at δH/δC 4.8/99 ppm (Figure 2B).^56^

The formate ester at δH/δC 8.0–8.5/161 ppm was present in the IFL as well as in the IML fraction (Figure 2B,C). However, no sharp signals indicating residual formic acid were observed. Model compounds treated with formic acid show that all free alcohols are esterified already after 30 min at 60 °C, and subsequent reactions are accelerated by increasing temperature (Figure S4). According to Ede and Brunow, the reactions of guaiacyl units include intramolecular cyclization, forming β-O-4’-(α-5′) 5-membered ring, and acid catalyzed dehydration followed by acidolysis.^36^ In IFL, the side-chain signals of β-O-4 were decreased compared to the other spectra (Table S1). However, no intramolecular cyclization product signals were found in HSQC, suggesting that, in addition to the high syringyl unit content, steric hindrance may also prevent this reaction in polymeric lignin. In contrast to IFL, the β-O-4 signals were preserved in the IML-fraction, possibly because of the slightly lower reflux temperature of the ethanol-formic acid mixture. The stability of formate esters during acetylation was revealed in ^13^C NMR, showing residual formate ester signals even after acetylation (Figures S5 and S6).

All samples contained wood extractives shown mainly at the aliphatic region (δH/δC, 3.5–0.5/40–15 ppm). Common extractives in poplar are fatty acid tri- and diglycerides, fatty acids (mostly C18:2), hydroxycinnamic acids, sterols, and corresponding fatty acid esters.^57^ A cluster of signals was present in all spectra at δH/δC 3.3–3.5/68–75 ppm, and because some of the signals had correlation to the aliphatic region in HMBC spectra (Figure S5), the cluster is likely to be extractives. Signals in this area are often interpreted as carbohydrates,^58^ but in acetylated samples, their signals should be shifted to δH/δC 4.5–5.5/65–75 ppm.^59^ Also, the peaks in the cluster δH/δC 3.3–3.5/68–75 ppm are sharp compared to broad polymeric signals, indicating small MW molecules. Small water-soluble sugars should have also been removed during pre-treatment washing of the wet lignin cake. Extractives instead could remain more easily with the lignin and dissolve into the extraction solvent, ending up in the soluble fractions.

The hydroxyl contents of extracted lignin fractions were evaluated using the ^31^P NMR technique (Table 2). The high hydroxyl content of lignin is a useful property for targeted lignin modification, as both phenolic and aliphatic hydroxyl functional groups can be functionalized.^6,60^ The highest amount of total hydroxyls was present in the IEL fraction (Table 2), which can be explained by the low MWD accompanied by a high amount of phenolic end groups. Compared to IEL, IBL had a similar aliphatic hydroxyl content but significantly lower phenolic hydroxyl content due to the relatively high MWD of this fraction, which had a *M*_w_ twice that of the IEL. All fractions contained high amounts (11–13%) of *p*-hydroxybenzoate ester groups, as shown by H-type phenolic hydroxyls that were not cleaved in either mild alkaline or acidic conditions.

**Table 2:** Hydroxyl Content of the Different Samples in mmol/g Measured by ^31^P NMR

fraction	aliphatic	5-subst^b^/S	G	H^a^	–COOH	S/G/H	total phenolic	total
IEL	2.69	2.04/1.04	1.11	0.40	0.27	1/1.07/0.38	3.55	6.51
IFL	0.91	1.38/0.53	0.55	0.27	0.22	1/1.04/0.51	2.20	3.33
IML	1.78	1.36/0.67	0.72	0.31	0.18	1/1.07/0.46	2.39	4.35
IBL	2.40	1.05/0.67	0.59	0.22	0.03	1/0.88/0.33	1.86	4.29

a Terminal p-benzoic acid esters; IEL 11.3%, IFL 12.3%, IML 13.0%, IBL 11.8% of total phenolic OHs.

b Total 5-substituted, includes syringyl phenols.

The formic acid soluble lignin fraction (IFL) had the lowest total hydroxyl content mainly because of reduced amounts of aliphatic hydroxyls, which are known to be substituted and modified by formic acid.^36^ Accordingly, IML had comparable total hydroxyl content, but higher phenolic and lower aliphatic hydroxyl content than IBL.

The number of condensed 5-substituted phenols seems unusually high in all fractionated samples, likely due to overlap of syringyl phenol signals with other 5-substituted phenols, biasing the results. HSQC integration (Table S1) suggests that the lignin fractions contain slightly more syringyl groups than guaiacyl groups, in contrast to the ^31^P NMR integration results.

All samples were analyzed with pyrolysis-GC-MS to evaluate structural alterations indicated by differences in the polymer fragmentation pattern. Identified lignin-based compounds from pyrolysis of soluble fractions and their relative abundances are listed in Table 3. The soluble fractions contained only small amounts of carbohydrate-derived fragments. IBL contained higher amounts of propyl side-chain structures (C3), indicating higher MW lignin with substantial amounts of β-O-4 ether structures.^61^ On the other hand, IEL contained lower MW polymers with a narrow polydispersity and contained mostly macromolecules from pentamers to decamers, indicating that 20–40% of the lignin phenylpropane units occurred as end-groups. Consequently, the abundance of C3 structures was substantially lower and the number of short units was higher than in IBL. Both IFL and IML fractions contained lower amounts of C3 structures than the other fractions, likely caused by structural modification of β-aryl ether structures by formic acid.

**Table 3:** Pyrolysis-GC-MS Base Peak Integrals of Known Lignin Fragments^a^

compounds	RT (min)	IBL	IEL	IML	IFL	SEL
phenol	7.20	12.04	40.03	53.16	30.14	40.57
2-methylphenol	8.50	0.72	0.88	1.19	1.92	2.11
4-methylphenol	8.61	0.46	0.67	1.01	1.90	2.42
2-methoxyphenol	8.94	20.25	29.08	24.93	38.19	29.55
2-methoxy-6-methylphenol	10.40	6.00	0.96	1.25	2.45	1.73
4-methyl-2-methoxyphenol	10.58	7.38	4.62	4.14	9.69	8.41
3,4-dimethoxytoluene	11.30	0.19	0.21	0.28	0.38	0.36
3-methoxy-1,2-benzenediol	11.46	0.63	0.02	0.01	0.12	0.31
4-ethyl-2-methoxyphenol	11.84	2.92	1.08	1.52	2.14	3.10
2-methoxy-4-vinylphenol	12.35	9.03	5.81	6.45	3.01	2.76
2,6-dimethoxyphenol	12.84	19.95	7.86	3.49	4.88	2.13
eugenol	12.91	0.87	0.61	0.51	0.34	0.48
2-methoxy-4-propylphenol	13.04	0.68	0.21	0.18	0.42	0.45
vanillin	13.62	1.19	1.31	0.10	0.39	0.52
*cis*-isoeugenol	13.65	0.55	0.24	0.18	0.14	0.09
2,6-dimethoxy-4-methylphenol	14.11	6.16	0.71	0.24	0.61	0.68
*trans*-isoeugenol	14.22	2.97	0.94	0.39	0.29	0.37
acetovanillone	14.62	0.64	0.54	0.06	0.15	0.36
1-(4-hydroxy-3-methoxyphenyl) 2-propanone	15.17	0.79	0.20	0.03	0.12	0.17
4-ethylsyringol	15.19	1.17	0.07	0.01	0.06	0.17
4-vinylsyringol	15.48	3.55	0.20	0.04	0.11	0.68
5-methoxyeugenol	16.04	0.82	0.04	0.01	0.03	0.17
*cis*-isomethoxyeugenol	16.42	0.27	0.02	0.00	0.01	0.08
syringaldehyde	16.55	0.01	1.82	0.42	1.26	1.15
*trans*-isomethoxyeugenol	17.18	0.72	0.12	0.03	0.11	0.60
acetosyringone	17.61	0.02	1.77	0.37	1.16	0.57
SUM		100	100	100	100	100
SUM C0–C2^b^		92.3	97.6	98.7	98.5	97.6
SUM C3^c^		7.7	2.4	1.3	1.5	2.4

a Analysis shows relative abundance of the most important lignin pyrolysis fragments. Abundances of compounds having side chains of less than three carbons (C0–C2) were added together and compared to the sum of compounds with three carbon (C3) side chains. RT shows the retention time of each fragmented compound.

b C0–C2 represents lignin fragments with 0–2 carbon aliphatic side chains.

c C3 represents lignin fragments with three carbon aliphatic side chains.

All the insoluble fractions, as well as the original SEL, contained a notable amount of carbohydrate components, as indicated by the pyrolysis products like furfurals, at the typical carbohydrate region (Figure S2).^53,61^ In the insoluble IFH and IMH fractions, lignin-originated volatile products were diminished compared to carbohydrate-based products, indicating higher amounts of carbohydrates in these samples. The chromatograms of the insoluble samples also agree with the IR spectra, indicating the presence of carbohydrates.

### 3.4 Thermal Analysis

**Figure 3:**
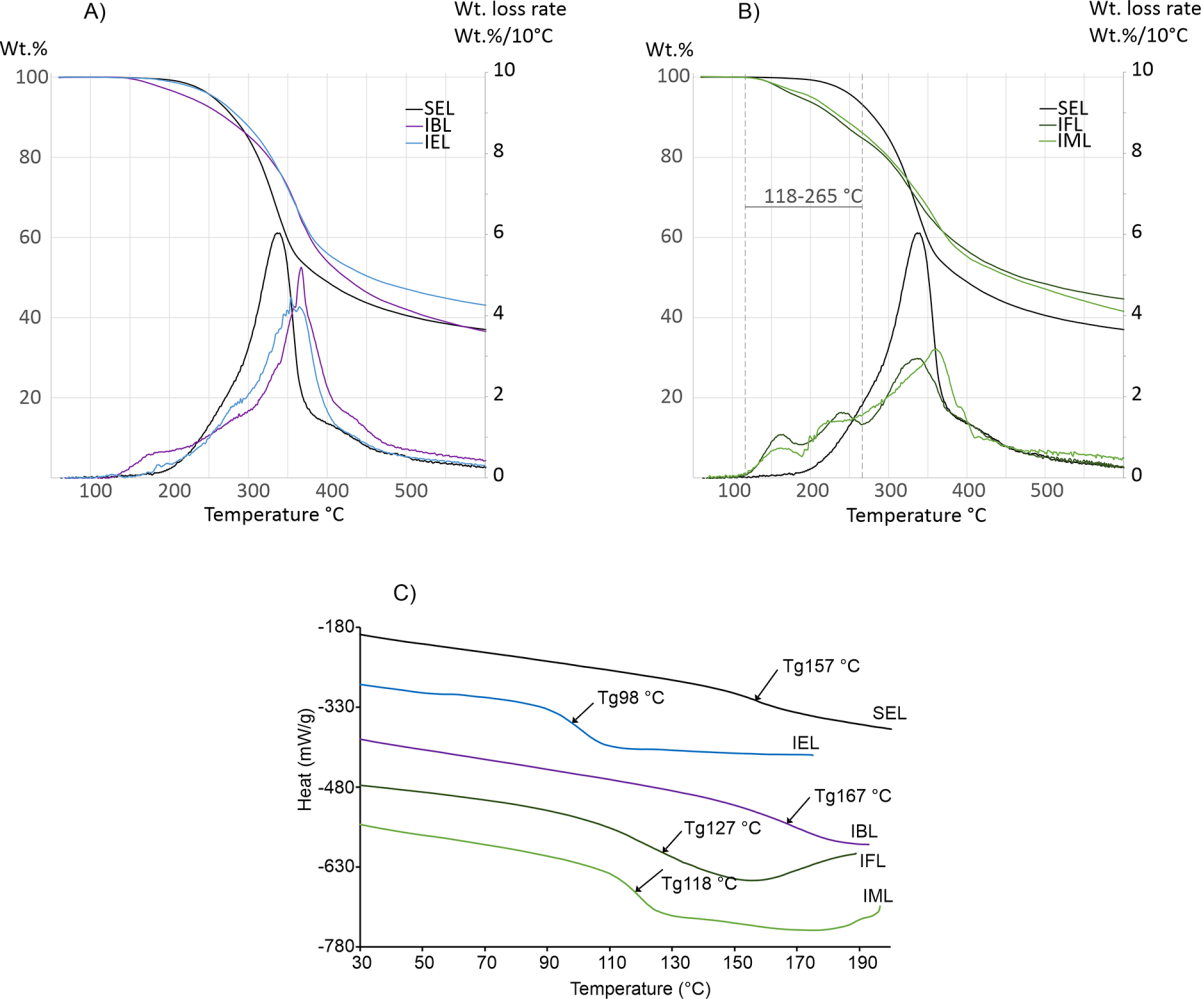
TG, DTG, and DSC thermograms. TG and DTG thermograms of (A) IBL and IEL and (B) IFL and IML; (C) DSC thermogram of soluble fractions. IFL and IML had a distinct thermal degradation pattern at 118–265 °C in comparison to IEL and IBL, suggesting reactions of formate esters at this temperature range.

The relative stabilities and decomposition of the starting material SEL and its four soluble fractions were studied with TGA. The thermograms are presented in Figure 3 and summarized in Table 4. According to literature, lignin thermal degradation typically occurs in a wide temperature range (150–500 °C)^62,63^ and consists of several stages.^62−64^ The first stage is the removal of residual hydrogen-bonded water slightly above 100 °C, followed by the aryl β-ether linkages at 230–260 °C and C–C linkage cleavage at 275–350 °C.^65^ The thermal stabilities were evaluated using the onset temperature, which is an extrapolated point describing the starting temperature for decomposition. The onset temperature of SEL was 282 °C, whereas IEL gave +13 °C and IBL +23 °C higher values (Figure 3A). Formic acid-extracted lignins (IFL and IML) both gave −45 and −30 °C lower values, due to thermally unstable formate groups in the polymers (Figure 3B).

**Table 4:** Thermal Data of Soluble Fractions and SEL

sample	*T*_g_ (°C)	Δ*T*_g_ (°C)	onset (°C)	DTG_max_ (°C)	residue (785 °C) (wt %)
SEL	157		282	337	34.0
IEL	98	–59	295	354	40.2
IFL	127	–30	305	331	41.8
IML	118	–39	237	361	35.3
IBL	167	+10	305	367	32.8

The obtained residual masses showed the amount of char leftover after heating the sample to 785 °C in a N_2_ atmosphere, and typically, lignins have residues of 40 wt % due to their aromatic structure.^66^ SEL had 34.0 wt % residual mass, which indicated that it also contained more carbohydrates.^67^ Most of the fractions had higher mass values except IBL (Figure 3A); for example, IEL had 40.2 wt % residual mass, which is 6.2 wt % higher than in the starting material (SEL).

The TGA and derivative thermogravimetric (DTG) curves of SEL, IEL, and IBL are illustrated in Figure 3A. The DTG curves were created from TGA curves to show a mass loss rate (wt %/min) and to visualize the major steps as partial or whole decompositions occurring during the heating process. The original SEL containing the highest amount of carbohydrates had its maximum decomposition rate (DTG_max_) at 337 °C (Table 4). The fractionated lignins IEL and IBL were shifted to higher, more lignin-like decomposition temperatures at 354 and 366 °C, respectively, which supports the absence of carbohydrates in the extracted fractions.

DSC was used to determine the glass transition temperatures (*T*_g_) of the different solubilized fractions. Lignins generally have a *T*_g_ between 90 and 180 °C depending on the isolation method, lignin structure, and MW.^68,69^ DSC measurement for SEL gave a *T*_g_ of 157 °C (Table 4) and NaOH-soluble lignin fraction (IBL) had slightly higher *T*_g_ (167 °C) than SEL. Both are in accordance with earlier findings for biorefinery technical lignins.^67^ IEL had typical low MW lignin *T*_g_ of 98 °C, resembling Alcell lignin, and this increases its processability and applicability for polymer blends.^70^

The formylated fractions IFL and IML have reduced hydrogen bonding leading to lower *T*_g_ compared to IBL, and only the first heating cycle was evaluated due to thermal degradation of the formate groups, as suggested by TGA at 118–265 °C (Figure 3B, Scheme S1). Therefore, the measurements were performed using a temperature program with three heating ramps from 30 to 210 °C (including anneal cycle at start) to better understand the phenomenon. For IFL, the first heat cycle gave a *T*_g_ of 127 °C, the second heat cycle a *T*_g_ of 162 °C, and the third heat cycle a *T*_g_ of 175 °C. The first heat cycle *T*_g_ of 127 °C is the most accurate value because it is not affected by formate decomposition. However, thermal history influences it to some degree. Acetyl substitution of lignin lowers *T*_g_ by approximately 22 °C.^60^ Formic acid treatment produces a similar effect, where the lignin structure gains mobility by occupation of hydroxyl groups by esters, thus preventing hydrogen bonding. The interesting chances in thermal behavior suggest lignin formylation to promote the formation of stimuli-responsive materials.

The thermal behavior of IML revealed differences between formic acid treated fractions, giving *T*_g_ values of 118, 126, and 137 °C for the first three heating cycles. This can be attributed to lower formylation degree, leading to decreased thermal degradation during the heating cycles. Previously, it has been shown that smaller degrees of acetylation (30 mol %) have almost the same effect on *T*_g_ as to full acetylation (100 mol %).^60^

Industrial production of the soluble fractions can be integrated in the current biorefinery concept, which requires more added-value products as outputs to increase its profitability. The closed cycle concept requires that all waste streams are minimized and ultimately eliminated. In this respect, the ethanol extraction method is particularly integrable because the solvent is produced from the cellulose fraction in the same biorefinery. Recycling of the solvents as a part of the process also increases its sustainability. The ultimate value of the process depends on the application potential of the soluble fractions. Reproducibility is a highly important factor in industrial applications, and the quality of all soluble fractions was reproducible. In addition, the IEL had particularly high solubility and dispersibility, which increases its applicability as a reinforcing filler in polymer blends, phenol replacement in phenol-formaldehyde resins, or as a polyol in polyurethane synthesis, for example.^7^ Knowledge of the characteristics and properties of these soluble fractions can accelerate further material development.

## 4 Conclusions

Biorefinery lignin residue (SEL) was extracted with ethanol, formic acid, and their mixture. All these treatments resulted in low MW fractions with very small carbohydrate content. Ethanol extraction gave the least modified lignin with high solubility, uniform polydispersity, and small MW at reasonable yields (33% of Klason) for enzymatic and chemical follow-up treatments. From a process integration perspective, ethanol is non-toxic, easily recyclable, and readily available from bioethanol processing. The formic acid-extracted lignin fractions were derivatized by formate esters and consequently could be used in various materials, such as in stimuli-responsive composite foams. This work provides essential knowledge on lignin fractionation for development of sophisticated lignin conversion technology and lignin-based materials.

## Supporting Information Available

Chemicals, base-treated lignin extraction protocol, *p-*hydroxybenzoic acid extraction protocol, GPC, lignin 1d and 2d NMR protocols, hydroxyl group determination by 31P-NMR, model compound NMR protocol, TGA protocol, DSC protocol, pyrolysis gas chromatography-mass spectrometry, lignin content by acetyl bromide assay protocol, FESEM protocol, gel permeation chromatograms, HPLC, pyrolysis-GC-MS, NMR spectral analysis, HSQC and HMBC of soluble lignin fraction IEL, 13C NMR of the fractionated lignins, 13C NMR integrations, HSQC-NMR integrals, scheme of lignin formylation and formate ester thermal degradation, and scanning electron microscopy imaging (PDF)

## References

[ref1] Manzanares P. The Role of Biorefinering Research in the Development of a Modern Bioeconomy. Acta Innovations 2020, 37, 47–56. 10.32933/ActaInnovations.37.4.

[ref2] Lange L.; Connor K. O.; Arason S.; Bundgård-Jørgensen U.; Canalis A.; Carrez D.; Gallagher J.; Gøtke N.; Huyghe C.; Jarry B.; Llorente P.; Marinova M.; Martins L. O.; Mengal P.; Paiano P.; Panoutsou C.; Rodrigues L.; Stengel D. B.; van der Meer Y.; Vieira H. Developing a Sustainable and Circular Bio-Based Economy in EU: By Partnering Across Sectors, Upscaling and Using New Knowledge Faster, and For the Benefit of Climate, Environment & Biodiversity, and People & Business. Front. Bioeng. Biotechnol. 2021, 8, 619066 10.3389/fbioe.2020.619066. 33553123 PMC7860146

[ref3] Isikgor F. H.; Becer C. R. Lignocellulosic Biomass: A Sustainable Platform for the Production of Bio-Based Chemicals and Polymers. Polym. Chem. 2015, 6, 4497–4559. 10.1039/c5py00263j.

[ref4] Sjöström E. Wood Chemistry; Elsevier: San Diego CA, 1993.

[ref5] Løhre C.; Kleinert M.; Barth T. Organosolv Extraction of Softwood Combined with Lignin-to-Liquid-Solvolysis as a Semi-Continuous Percolation Reactor. Biomass Bioenergy 2017, 99, 147–155. 10.1016/j.biombioe.2017.02.014.

[ref6] Schutyser W.; Renders T.; Van den Bosch S.; Koelewijn S.-F.; Beckham G. T.; Sels B. F. Chemicals from Lignin: An Interplay of Lignocellulose Fractionation, Depolymerisation, and Upgrading. Chem. Soc. Rev. 2018, 47, 852–908. 10.1039/C7CS00566K. 29318245

[ref7] Doherty W. O. S.; Mousavioun P.; Fellows C. M. Value-Adding to Cellulosic Ethanol: Lignin Polymers. Ind. Crops Prod. 2011, 33, 259–276. 10.1016/j.indcrop.2010.10.022.

[ref8] Tripathi N.; Hills C. D.; Singh R. S.; Atkinson C. J. Biomass Waste Utilisation in Low-Carbon Products: Harnessing a Major Potential Resource. npj Clim. Atmos. Sci. 2019, 2, 35 10.1038/s41612-019-0093-5.

[ref9] Avellar B. K.; Glasser W. G. Steam-Assisted Biomass Fractionation. I. Process Considerations and Economic Evaluation. Biomass Bioenergy 1998, 14, 205–218. 10.1016/S0961-9534(97)10043-5.

[ref10] Jönsson L. J.; Martín C. Pretreatment of Lignocellulose: Formation of Inhibitory by-Products and Strategies for Minimizing Their Effects. Bioresour. Technol. 2016, 199, 103–112. 10.1016/j.biortech.2015.10.009. 26482946

[ref11] da Costa Lopes A. M. Biomass Delignification with Green Solvents towards Lignin Valorisation: Ionic Liquids vs Deep Eutectic Solvents. Acta Innovations 2021, 40, 64–78. 10.32933/ActaInnovations.40.5.

[ref12] Li J.; Gellerstedt G.; Toven K. Steam Explosion Lignins; Their Extraction, Structure and Potential as Feedstock for Biodiesel and Chemicals. Bioresour. Technol. 2009, 100, 2556–2561. 10.1016/j.biortech.2008.12.004. 19157871

[ref13] Dodd A. P.; Kadla J. F.; Straus S. K. Characterization of Fractions Obtained from Two Industrial Softwood Kraft Lignins. ACS Sustainable Chem. Eng. 2015, 3, 103–110. 10.1021/sc500601b.

[ref14] Jin J.; Ding J.; Klett A.; Thies M. C.; Ogale A. A. Carbon Fibers Derived from Fractionated-Solvated Lignin Precursors for Enhanced Mechanical Performance. ACS Sustainable Chem. Eng. 2018, 6, 14135–14142. 10.1021/acssuschemeng.8b02697.

[ref15] Zhang Y.; Hou Q.; Fu Y.; Xu C.; Smeds A. I.; Willför S.; Wang Z.; Li Z.; Qin M. One-Step Fractionation of the Main Components of Bamboo by Formic Acid-Based Organosolv Process Under Pressure. J. Wood Chem. Technol. 2018, 38, 170–182. 10.1080/02773813.2017.1388823.

[ref16] Wang Y. Y.; Li M.; Wyman C. E.; Cai C. M.; Ragauskas A. J. Fast Fractionation of Technical Lignins by Organic Cosolvents. ACS Sustainable Chem. Eng. 2018, 6, 6064–6072. 10.1021/acssuschemeng.7b04546.

[ref17] Klett A. S.; Payne A. M.; Thies M. C. Continuous-Flow Process for the Purification and Fractionation of Alkali and Organosolv Lignins. ACS Sustainable Chem. Eng. 2016, 4, 6689–6694. 10.1021/acssuschemeng.6b01560.

[ref18] Li J.; Henriksson G.; Gellerstedt G. Lignin Depolymerization/Repolymerization and Its Critical Role for Delignification of Aspen Wood by Steam Explosion. Bioresour. Technol. 2007, 98, 3061–3068. 10.1016/j.biortech.2006.10.018. 17141499

[ref19] Dastpak A.; Lourencon T. v.; Balakshin M.; Farhan Hashmi S.; Lundström M.; Wilson B. P. Solubility Study of Lignin in Industrial Organic Solvents and Investigation of Electrochemical Properties of Spray-Coated Solutions. Ind. Crops Prod. 2020, 148, 112310 10.1016/j.indcrop.2020.112310.

[ref20] Bokhary A.; Leitch M.; Liao B. Q. Liquid–Liquid Extraction Technology for Resource Recovery: Applications, Potential, and Perspectives. J. Water Proc. Eng. 2021, 40, 101762 10.1016/j.jwpe.2020.101762.

[ref21] Sadeghifar H.; Wells T.; Le R. K.; Sadeghifar F.; Yuan J. S.; Jonas Ragauskas A. Fractionation of Organosolv Lignin Using Acetone:Water and Properties of the Obtained Fractions. ACS Sustainable Chem. Eng. 2017, 5, 580–587. 10.1021/acssuschemeng.6b01955.

[ref22] Gioia C.; Lo Re G.; Lawoko M.; Berglund L. Tunable Thermosetting Epoxies Based on Fractionated and Well-Characterized Lignins. J. Am. Chem. Soc. 2018, 140, 4054–4061. 10.1021/jacs.7b13620. 29498848

[ref23] Rahimi A.; Azarpira A.; Kim H.; Ralph J.; Stahl S. S. Chemoselective Metal-Free Aerobic Alcohol Oxidation in Lignin. J. Am. Chem. Soc. 2013, 135, 6415–6418. 10.1021/ja401793n. 23570328

[ref24] Rahimi A.; Ulbrich A.; Coon J. J.; Stahl S. S. Formic-Acid-Induced Depolymerization of Oxidized Lignin to Aromatics. Nature 2014, 515, 249–252. 10.1038/nature13867. 25363781

[ref25] Nousiainen P.; Kontro J.; Maijala P.; Uzan E.; Hatakka A.; Lomascolo A.; Sipilä J. Lignin Model Compound Studies to Elucidate the Effect of “Natural” Mediators on Oxidoreductase-Catalyzed Degradation of Lignocellulosic Materials. ACS Symp. Ser. 2012, 1107, 229–242. 10.1021/bk-2012-1107.ch012.

[ref26] Adler E. Lignin Chemistry-Past, Present and Future. Wood Sci. Technol. 1977, 11, 169–218. 10.1007/BF00365615.

[ref27] Maijala P.; Mattinen M. L.; Nousiainen P.; Kontro J.; Asikkala J.; Sipilä J.; Viikari L. Action of Fungal Laccases on Lignin Model Compounds in Organic Solvents. J. Mol. Catal. B: Enzym. 2012, 76, 59–67. 10.1016/j.molcatb.2011.12.009.

[ref28] Sipponen M. H.; Lange H.; Crestini C.; Henn A.; Österberg M. Lignin for Nano- and Microscaled Carrier Systems: Applications, Trends, and Challenges. ChemSusChem 2019, 12, 2039–2054. 10.1002/cssc.201900480. 30933420 PMC6593669

[ref29] Hyväkkö U.; Maltari R.; Kakko T.; Kontro J.; Mikkilä J.; Kilpeläinen P.; Enqvist E.; Tikka P.; Hildén K.; Nousiainen P.; Sipilä J. On the Effect of Hot-Water Pretreatment in Sulfur-Free Pulping of Aspen and Wheat Straw. ACS Omega 2020, 5, 265–273. 10.1021/acsomega.9b02619. 31956773 PMC6964294

[ref30] Kontro J.; Lyra C.; Koponen M.; Kuuskeri J.; Kähkönen M. A.; Wallenius J.; Wan X.; Sipilä J.; Mäkelä M. R.; Nousiainen P.; Hildén K. Production of Recombinant Laccase From Coprinopsis Cinerea and Its Effect in Mediator Promoted Lignin Oxidation at Neutral PH. Front. Bioeng. Biotechnol. 2021, 9, 767139 10.3389/fbioe.2021.767139. 34858962 PMC8630700

[ref31] Kontro J.; Maltari R.; Mikkilä J.; Kähkönen M.; Mäkelä M. R.; Hildén K.; Nousiainen P.; Sipilä J. Applicability of Recombinant Laccases From the White-Rot Fungus Obba Rivulosa for Mediator-Promoted Oxidation of Biorefinery Lignin at Low PH. Front. Bioeng. Biotechnol. 2020, 8, 604497 10.3389/fbioe.2020.604497. 33392170 PMC7773891

[ref32] Wallenius J.; Kontro J.; Lyra C.; Kuuskeri J.; Wan X.; Kähkönen M. A.; Baig I.; Kamer P. C. J.; Sipilä J.; Mäkelä M. R.; Nousiainen P.; Hildén K. Depolymerization of Biorefinery Lignin by Improved Laccases of the White-rot Fungus Obba Rivulosa. Microb. Biotechnol. 2021, 14, 2140–2151. 10.1111/1751-7915.13896. 34310858 PMC8449659

[ref33] Chum H. L.; Ratcliff M.; Schroeder H. A.; Sopher D. W. Electrochemistry of Biomass-Derived Materials I. Characterization, Fractionation, and Reductive Electrolysis of Ethanol-Extracted Explosively-Depressurized Aspen Lignin. J. Wood Chem. Technol. 1984, 4, 505–532. 10.1080/02773818408070665.

[ref34] Routray W.; Orsat V. Microwave-Assisted Extraction of Flavonoids: A Review. Food Bioprocess Technol. 2012, 5, 409–424. 10.1007/s11947-011-0573-z.

[ref35] Ede R. M.; Brunow G. Formic Acid/Peroxyformic Acid Pulping. III. Condensation Reactions of ß-Aryl Ether Model Compounds in Formic Acid. Holzforschung 1989, 43, 317–322. 10.1515/hfsg.1989.43.5.317.

[ref36] Ede R.; Brunow G.; Poppius K.; Sundquist J.; Hortling B. Formic Acid/Peroxyformic Acid Pulping. Nord. Pulp Pap. Res. J. 1988, 3, 119–123. 10.3183/npprj-1988-03-03-p119-124.

[ref37] Martin-Sampedro R.; Capanema E. A.; Hoeger I.; Villar J. C.; Rojas O. J. Lignin Changes after Steam Explosion and Laccase-Mediator Treatment of Eucalyptus Wood Chips. J. Agric. Food Chem. 2011, 59, 8761–8769. 10.1021/jf201605f. 21749069

[ref38] Wang G.; Chen H. Fractionation and Characterization of Lignin from Steam-Exploded Corn Stalk by Sequential Dissolution in Ethanol-Water Solvent. Sep. Purif. Technol. 2013, 120, 402–409. 10.1016/j.seppur.2013.10.029.

[ref39] Rashid T.; Sher F.; Rasheed T.; Zafar F.; Zhang S.; Murugesan T. Evaluation of Current and Future Solvents for Selective Lignin Dissolution–A Review. J. Mol. Liq. 2021, 321, 114577 10.1016/j.molliq.2020.114577.

[ref40] Kumar A. K.; Sharma S. Recent Updates on Different Methods of Pretreatment of Lignocellulosic Feedstocks: A Review. Bioresour. Bioprocess. 2017, 4, 7 10.1186/s40643-017-0137-9. 28163994 PMC5241333

[ref41] Sannigrahi P.; Ragauskas A. J.; Tuskan G. A. Poplar as a Feedstock for Biofuels: A Review of Compositional Characteristics. Biofuels, Bioprod. Biorefin. 2010, 4, 209–226. 10.1002/bbb.206.

[ref42] Jablonsky M.; Haz A.; Orságová A.; Botková M.; Šmatko L.; Kočiš J. Relationships between Elemental Carbon Contents and Heating Values of Lignins. 4th International Conference on Renewable Energy Sources & Energy Efficiency, 2013; Vol. 358, pp 67–72.

[ref43] Jablonsky M.; Botkova M.; Adamovska J. Prediction of Methoxyl Groups Content in Lignin Based on Ultimate Analysis. Cellul. Chem. Technol. 2015, 49, 165–168.

[ref44] Lin S. Y.; Dence C. W. Methods in Lignin Chemistry; Dence C. W., Lin S. Y., Timell T. E., Eds.; Springer Series in Wood Science; Springer: Berlin, 1992.

[ref45] Mörck R.; Yoshida H.; Kringstad K. P.; Hatakeyama H. Fractionation of Kraft Lignin by Successive Extraction with Organic Solvents I. Functional Groups, 13C-NMR Spectra and Molecular Weight Distributions. Holzforschung 1986, 40, 51–60. 10.1515/hfsg.1988.42.2.111.

[ref46] Cao S.; Pu Y.; Studer M.; Wyman C.; Ragauskas A. J. Chemical Transformations of Populus Trichocarpa during Dilute Acid Pretreatment. RSC Adv. 2012, 2, 10925–10936. 10.1039/c2ra22045h.

[ref47] Baumberger S.; Abaecherli A.; Fasching M.; Gellerstedt G.; Gosselink R.; Hortling B.; Li J.; Saake B.; de Jong E. Molar Mass Determination of Lignins by Size-Exclusion Chromatography: Towards Standardisation of the Method. Holzforschung 2007, 61, 459–468. 10.1515/HF.2007.074.

[ref48] Raspolli Galletti A. M.; D’Alessio A.; Licursi D.; Antonetti C.; Valentini G.; Galia A.; Nassi o Di Nasso N. Midinfrared FT-IR as a Tool for Monitoring Herbaceous Biomass Composition and Its Conversion to Furfural. J. Spectrosc. 2015, 2015, 719042 10.1155/2015/719042.

[ref49] Popescu C. M.; Popescu M. C.; Singurel G.; Vasile C.; Argyropoulos D. S.; Willfor S. Spectral Characterization of Eucalyptus Wood. Appl. Spectrosc. 2007, 61, 1168–1177. 10.1366/000370207782597076. 18028695

[ref50] Schwanninger M.; Rodrigues J. C.; Pereira H.; Hinterstoisser B. Effects of Short-Time Vibratory Ball Milling on the Shape of FT-IR Spectra of Wood and Cellulose. Vib. Spectrosc. 2004, 36, 23–40. 10.1016/j.vibspec.2004.02.003.

[ref51] Tejado A.; Peña C.; Labidi J.; Echeverria J. M.; Mondragon I. Physico-Chemical Characterization of Lignins from Different Sources for Use in Phenol-Formaldehyde Resin Synthesis. Bioresour. Technol. 2007, 98, 1655–1663. 10.1016/j.biortech.2006.05.042. 16843657

[ref52] Ralph J.; Landucci L. NMR of Lignins. Lignin and Lignans; CRC Press, 2010; pp 137–243.

[ref53] Constant S.; Wienk H. L. J.; Frissen A. E.; de Peinder P.; Boelens R.; van Es D. S.; Grisel R. J. H.; Weckhuysen B. M.; Huijgen W. J. J.; Gosselink R. J. A.; Bruijnincx P. C. A. New Insights into the Structure and Composition of Technical Lignins: A Comparative Characterisation Study. Green Chem. 2016, 18, 2651–2665. 10.1039/c5gc03043a.

[ref54] Bichot A.; Lerosty M.; Geirnaert L.; Méchin V.; Carrère H.; Bernet N.; Delgenès J. P.; García-Bernet D. Soft Microwave Pretreatment to Extract P-Hydroxycinnamic Acids from Grass Stalks. Molecules 2019, 24, 3885 10.3390/molecules24213885. 31661930 PMC6864740

[ref55] Kim H.; Ralph J. Solution-State 2D NMR of Ball-Milled Plant Cell Wall Gels in DMSO-d 6/Pyridine-D5. Org. Biomol. Chem. 2010, 8, 576–591. 10.1039/b916070a. 20090974 PMC4070321

[ref56] Hedenström M.; Wiklund-Lindström S.; Öman T.; Lu F.; Gerber L.; Schatz P.; Sundberg B.; Ralph J. Identification of Lignin and Polysaccharide Modifications in Populus Wood by Chemometric Analysis of 2D NMR Spectra from Dissolved Cell Walls. Mol. Plant 2009, 2, 933–942. 10.1093/mp/ssp047. 19825670

[ref57] Xu C.; Qin M.; Fu Y.; Liu N.; Hemming J.; Holmbom B.; Willför S. Lipophilic Extractives in Populus Euramericana Guariento Stemwood and Bark. J. Wood Chem. Technol. 2010, 30, 105–117. 10.1080/02773810903085994.

[ref58] Wang X.; Hou Q.; Zhang X.; Zhang Y.; Liu W.; Xu C.; Zhang F. Color Evolution of Poplar Wood Chips and Its Response to Lignin and Extractives Changes in Autohydrolysis Pretreatment. Int. J. Biol. Macromol. 2020, 157, 673–679. 10.1016/j.ijbiomac.2019.11.224. 31794829

[ref59] Rencoret J.; Pereira A.; del Río J. C.; Martínez A. T.; Gutiérrez A. Laccase-Mediator Pretreatment of Wheat Straw Degrades Lignin and Improves Saccharification. BioEnergy Res. 2016, 9, 917–930. 10.1007/s12155-016-9745-z.

[ref60] Koivu K. A. Y.; Sadeghifar H.; Nousiainen P. A.; Argyropoulos D. S.; Sipilä J. Effect of Fatty Acid Esterification on the Thermal Properties of Softwood Kraft Lignin. ACS Sustainable Chem. Eng. 2016, 4, 5238–5247. 10.1021/acssuschemeng.6b01048.

[ref61] Dizhbite T.; Telysheva G.; Dobele G.; Arshanitsa A.; Bikovens O.; Andersone A.; Kampars V. Py-GC/MS for Characterization of Non-Hydrolyzed Residues from Bioethanol Production from Softwood. J. Anal. Appl. Pyrolysis 2011, 90, 126–132. 10.1016/j.jaap.2010.11.004.

[ref62] Khongchamnan P.; Wanmolee W.; Laosiripojana N.; Champreda V.; Suriyachai N.; Kreetachat T.; Sakulthaew C.; Chokejaroenrat C.; Imman S. Solvothermal-Based Lignin Fractionation From Corn Stover: Process Optimization and Product Characteristics. Front. Chem. 2021, 9, 697237 10.3389/fchem.2021.697237. 34422761 PMC8374146

[ref63] Toledano A.; Serrano L.; Garcia A.; Mondragon I.; Labidi J. Comparative Study of Lignin Fractionation by Ultrafiltration and Selective Precipitation. Chem. Eng. J. 2010, 157, 93–99. 10.1016/j.cej.2009.10.056.

[ref64] Lagerquist L.; Pranovich A.; Sumerskii I.; von Schoultz S.; Vähäsalo L.; Willför S.; Eklund P. Structural and Thermal Analysis of Softwood Lignins from a Pressurized Hot Water Extraction Biorefinery Process and Modified Derivatives. Molecules 2019, 24, 335 10.3390/molecules24020335. 30669257 PMC6359013

[ref65] Sebestyén Z.; Jakab E.; May Z.; Sipos B.; Réczey K. Thermal Behavior of Native, Washed and Steam Exploded Lignocellulosic Biomass Samples. J. Anal. Appl. Pyrolysis 2013, 101, 61–71. 10.1016/j.jaap.2013.02.011.

[ref66] Harman-Ware A. E.; Crocker M.; Pace R. B.; Placido A.; Morton S.; DeBolt S. Characterization of Endocarp Biomass and Extracted Lignin Using Pyrolysis and Spectroscopic Methods. BioEnergy Res. 2015, 8, 350–368. 10.1007/s12155-014-9526-5.

[ref67] Sameni J.; Krigstin S.; Santos Rosa D.; dos S.; Leao A.; Sain M. Thermal Characteristics of Lignin Residue from Industrial Processes. Bioresources 2014, 9, 725–737. 10.15376/biores.9.1.725-737.

[ref68] Michelin M.; Liebentritt S.; Vicente A. A.; Teixeira J. A. Lignin from an Integrated Process Consisting of Liquid Hot Water and Ethanol Organosolv: Physicochemical and Antioxidant Properties. Int. J. Biol. Macromol. 2018, 120, 159–169. 10.1016/j.ijbiomac.2018.08.046. 30102983

[ref69] Kubo S.; Kadla J. F. Poly(Ethylene Oxide)/Organosolv Lignin Blends: Relationship between Thermal Properties, Chemical Structure, and Blend Behavior. Macromolecules 2004, 37, 6904–6911. 10.1021/ma0490552.

[ref70] Kun D.; Pukánszky B. Polymer/Lignin Blends: Interactions, Properties, Applications. Eur. Polym. J. 2017, 93, 618–641. 10.1016/j.eurpolymj.2017.04.035.

