# Supplementary material for: Fractionation of Technical Lignin from Enzymatically Treated Steam-Exploded Poplar Using Ethanol and Formic Acid
Source: ACS Appl Polym Mater. 2022 Nov 24;4(12):9388–98. doi: 10.1021/acsapm.2c01665 (PMC9748740; doi:10.1021/acsapm.2c01665)
Supplement: Supplementary file 1 — ap2c01665_si_001.pdf [file ap2c01665_si_001.pdf]

SUPPLEMENTARY INFORMATION

Fractionation of technical lignin from enzymatically treated steam exploded poplar using ethanol and formic acid

Riku Maltari[†,⸸], Jussi Kontro[†], Klaus Koivu[†], Muhammad Farooq[§], Joona Mikkilä[†,⸸], Rui Zhang[†], Kristiina Hildén[⸸], Jussi Sipilä[†], Paula A. Nousiainen[†,§,*]

[†] Department of Chemistry, University of Helsinki, FI-00014, P.O. Box 55, A. I. Virtasen Aukio 1, Helsinki, Finland
[⸸]Department of Microbiology, University of Helsinki, FI-00014, P.O. Box 56, Viikinkaari 9, Helsinki, Finland
[§]Department of Bioproducts and Biosystems, Aalto University, FI-02150, Vuorimiehentie 1, Espoo, Finland
* Corresponding author e-mail: paula.nousiainen@helsinki.fi, paula.nousiainen@aalto.fi

Contents

Supporting materials and methods....S2
- Chemicals....S2
- Base-treated lignin extraction protocol....S2
- *p*-Hydroxybenzoic acid extraction protocol....S2
- Gel permeation chromatography (GPC)....S2
- Lignin 1d and 2d NMR protocols....S2
- Hydroxyl group determination by ^{31}P-NMR....S3
- Model compound NMR protocol....S3
- Thermogravimetric analysis (TGA) protocol....S3
- Differential scanning calorimetry (DSC) protocol....S4
- Pyrolysis gas chromatography-mass spectrometry....S4
- Lignin content by acetyl bromide assay protocol....S4
- Field emission scanning electron microscopy (FESEM) protocol....S4

Gel permeation chromatograms....S5
High performance liquid chromatography (HPLC)....S6
Pyrolysis-GC-MS....S7
Nuclear magnetic resonance (NMR) spectral analysis of lignin model compound treated by formic acid....S8
HSQC and HMBC of soluble lignin fraction IEL....S12
^{13}C NMR of the fractionated lignins....S13
^{13}C NMR integrations of Acetate and Formate carbonyl regions....S15
HSQC-NMR integrals of major lignin aromatic groups and side-chain regions....S17
Scheme of lignin formylation and formate ester thermal degradation....S18
Scanning electron microscope imaging of the isolated lignins....S19
References....S20

Supporting materials and methods

Chemicals

n-Hexane (Honeywell, Riedel-de-Haën, HPLC, 97%), CHCl3 (Honeywell, Riedel-de-Haën, HPLC, 99.8%), sulphanilamide (Aldrich >99%), Acetyl bromide (Aldrich, 99%), NaOH (VWR AnalaR 99.1%), H2SO4 conc (VWR 95%), EtOAc (Honeywell, Riedel-de-Haën, HPLC, 99.7%), Na_2SO_4 anhydrous, Fischer Chemical, 99%)), EtOH (VWR, 96%), pyridine (VWR, AnalaR, 100%), Acetic anhydride (Ac_2O, VWR, AnalaR 96.3%), toluene (VWR, HPLC, 100%), THF (VWR, stabilized HPLC 99.8%), polystyrene standards (Scientific Polymer Products and Fluka Analytical), DMSO-d6 (Eurisotop, 99.8%D), CDCl3 (Eurisotop, 99.8%D), endo-N-hydroxy-5-norbornene-2,3-dicarboxylic acid imide (e-NHI, Aldrich 97%), chromium(III) acetylacetonate (Cr(acac)3, Aldrich, 99.99%), 2-chloro-4,4,5,5-tetramethyl-1,3,2-dioxaphospholane (Aldrich 95%), Acetone-d6 (Eurisotop, 99.8%D), 1-(3,4-Dimethoxyphenyl)-2-(2-methoxyphenoxy)-propane-1,3-diol (Adlerol, synthesized according to Nakatsubo et al. (1975)[1])

Base-treated lignin extraction protocol

Approximately 3 g of washed dry lignin was mixed in 70 ml of 0.1 M NaOH-solution for 2 hours at 60°C. The solids were filtered out and the lignin was precipitated out of solution by adjusting the pH to 2 with 2 M H_2SO_4. The precipitated lignin was filtered out of solution and washed with distilled water until the pH was 5. The wet base-treated lignin dried in ambient temperature and pressure for one week.

p-Hydroxybenzoic acid extraction protocol

The p-hydroxybenzoic acid content of SEL was determined after alkaline hydrolysis. SEL (1g) was immersed in 2 M NaOH solution, placed in silicon carbide vessel and heated in a microwave reactor (Anton Paar Monowave 450) at 110° C for 30 minutes. The resulting slurry was filtered, and the filtrate was acidified to pH 2 with 1 M sulfuric acid. The precipitated lignin was filtered out. The solid and liquid phases were extracted with 3x30 ml ethyl acetate. The ethyl acetate phase was separated from the water phase and dried with brine and anhydrous Na2SO4. Ethyl acetate was evaporated into dryness and the extract yield was 7%. The sample was analyzed by HPLC 1260 Infinity II (Agilent) with 6120 single quadrupole LC-MS equipped with autosampler. Presence of p-hydroxybenzoic acid as main component was verified using pure compound as a standard sample and shown as detailed in supplementary information Figure S2.

Gel permeation chromatography (GPC)

The samples were acetylated for NMR and GPC experiments, by mixing 50–100 mg of isolated lignin with 1 ml of pyridine and 1 ml of acetic anhydride overnight. The acetylated samples were isolated by evaporating the samples to dryness with ethanol (96%) and toluene (99%). The GPC samples were prepared by dissolving the acetylated samples 1 mg/ml in THF (Sigma-Aldrich) and filtering the dissolved samples through 0.2 µm Acrodisc GHP membrane HPLC filters (Waters). The IEL and IBL samples passed through the filters entirely. The IFL and IML samples had some residue remaining in the filter. The GPC samples were run in 1260 Infinity HPLC (Agilent) with Acquity APC XT 200 2.5 µm and 45 1.7 µm columns (Waters) at 40°C with 0.8 ml/min THF elution. The detection was performed at 280 nm with UV and refractive index (RI) detector. The molar mass was calibrated with the use of polystyrene standards (Scientific Polymer Products and Fluka Analytical). ChemStation GPC add-on was applied for data processing to obtain Mn (number average molecular weight), Mw (weight average molecular weight) and PD (polydispersity) values.

Lignin 1d and 2d NMR protocols

The 1H-13C correlation experiment was a heteronuclear single quantum correlation experiment (HSQC) (Bruker standard NMR pulse program “hsqcetgpsisp2.2”). The experiment was conducted using 32 scans, 16 dummy scans, 256 data points collected in the F1 dimension, with spectral windows of 6356 Hz in the F2 dimension and 27040 Hz in the F1 dimension. The transmitter frequency offsets were 2338 Hz in the F2 dimension and 12576 Hz in the F1 dimension. The interscan delay was 1.44 s and the acquisition time was

80 ms. Total acquisition time was 3 hours and 30 minutes. The pulse sequence of hmbcqplndqf for heteronuclear multiple bond correlation (HMBC) were used in acquisition with 11.8- and 210-ppm spectral widths in F2(1H) and F1 (13C), respectively. The 13C-NMR experiments were conducted using standard Bruker NMR pulse program, zgtg using 10000 scans with a spectral width of 27574 Hz and a transmitter frequency offset of 12576 Hz. The interscan delay was 1.5 seconds and the acquisition time was 1.2 seconds. The total acquisition time was approximately 10 hours. The spectra were analyzed with Bruker Topspin 4.0.5 Windows version using standard processing parameters.

Hydroxyl group determination by ^{31}P-NMR

Accurately weighed amount (40 mg) of lignin was dissolved in 0.6 ml of 1.6:1 pyridine:chloroform-d mixture. N-hydroxy-5-norborene-2,3-dicarboxylic acid imide (e-NHI) as an internal standard was dissolved in chloroform-d (9.27 mg/ml) and 200 µl of this solution was added to the dissolved sample. Also, 50 µl of 5.6 mg/ml Cr(AcAc)3 in chloroform-d was added to the mixture. Right before the measurement was conducted, 150 µl of phosphitylation reagent 2-chloro-4,4,5,5-tetramethyl-1,3,2-dioxaphospholane was added to the sample. Over the course of approximately 15 minutes, 200 scans were acquired in 31P using a 90° pulse flip angle. Chemical shifts were referenced to the phosphitylated water at 132.2 ppm in CDCl3. Different regions were integrated and compared to the integral of the internal standard at 152.0–151.8. The regions were 149.2–145.0 for aliphatic hydroxyls, 143.0–142.4 for syringyl phenolic hydroxyls, 140.4–138.5 for guaiacyl phenolic hydroxyls, 138.5–137.2 p-hydroxyphenyl phenolic hydroxyls and 136.0–133.9 for carboxylic acids.

Model compound NMR protocol

The acetylated samples were dissolved in d6-acetone and measured using Bruker Avance III 500 MHz NMR spectrometer with a BBFO broad band probe. The residual solvent peak (δC 29.50 and δH 2.05) was used as an internal reference. The 1H-13C correlation experiment was a heteronuclear single quantum correlation experiment (HSQC) (Bruker standard NMR pulse program “hsqcetgp”). The experiment was conducted using 8 scans, 16 dummy scans, 128 data points collected in the F1 dimension, with spectral windows of 6356 Hz in the F2 dimension and 27040 Hz in the F1 dimension. The transmitter frequency offsets were 2338 Hz in the F2 dimension and 12576 Hz in the F1 dimension. The interscan delay was 1.44 seconds and the acquisition time was 80 ms. Total acquisition time was 26 minutes.

The 1H-13C total correlation experiment was a heteronuclear single quantum correlation – total correlation spectroscopy experiment (HSQC-TOCSY) (Bruker standard NMR pulse program “hsqcetgpml”). The experiment was conducted using 8 scans, 16 dummy scans, 128 data points collected in the F1 dimension, with spectral windows of 6010 Hz in the F2 dimension and 27040 Hz in the F1 dimension. The transmitter frequency offsets were 2338 Hz in the F2 dimension and 12576 Hz in the F1 dimension. The interscan delay was 1.44 seconds and the acquisition time was 85 ms. Total acquisition time was 28 minutes.

The 1H-13C multiple bond correlation experiment was a heteronuclear multiple bond correlation experiment (HMBC) (Bruker standard NMR pulse program “hmbcgpl2ndqf”). The experiment was conducted using 8 scans, 16 dummy scans, 128 data points collected in the F1 dimension, with spectral windows of 6010 Hz in the F2 dimension and 27040 Hz in the F1 dimension. The transmitter frequency offsets were 2338 Hz in the F2 dimension and 12576 Hz in the F1 dimension. The interscan delay was 1.44 seconds and the acquisition time was 85 ms. Total acquisition time was 28 minutes.

Thermogravimetric analysis (TGA) protocol

Raw data was exported from STARe Evaluation Window and imported to MS Excel from which both TGA and derivative TGA curves (α = 0.1) where formed. Sample sizes were 11–16 mg in reusable 70 µl alumina crucibles with lids. The temperature program used a ramp speed of 10°C/min and nitrogen flow of 50 ml/min and consisted of: (annealing cycle) ramp to 105°C, isothermal 20 minutes, ramp to 60°C, isothermal 2 minutes, (measurement cycle) ramp to 800°C. The results provided insight into the relative stability of the lignin fractions and the decomposition behavior of two formylated lignins.

Differential scanning calorimetry (DSC) protocol

Raw data was exported from TA Universal Analysis and imported to MS Excel from which the DSC curves were created. Sample sizes were 10–16 mg in aluminum hermetic pans with two drilled holes (diameter 0.28 mm). Temperature program used a ramp speed of 10°C/min and nitrogen flow of 50 ml/min: (annealing cycle) ramp to 105°C, isothermal 20 min, ramp to 20°C, (first heating cycle to remove thermal history) ramp to 190, ramp back to 20°C, and (second heating cycle used as measurement cycle) ramp to 220°C. The T_g for lignin samples was defined as one-half the change in heat capacity occurring over the transition. T_g was measured from the second heating cycle, except for formylated fractions (IFL and IML) where T_g was measured from the first heating cycle. The IBL was ball-milled for 20 minutes before measurement, to break down colloidal lignin particles formed in lignin precipitation.

Pyrolysis gas chromatography-mass spectrometry

All samples were dried thoroughly prior to analysis. The pyrolysis was performed under the same conditions for all samples. Py–GC-MS equipment Pyrolab2000 (Pyrolab, Sweden) with a platinum foil pulse pyrolyzer and 580°C isothermal pyrolysis temperature was used for pyrolysis. The system was directly connected to Bruker Scion SQ 456-GC/MS equipped with Agilent DB-5MS UI (5%-(phenyl)-methylpolysiloxane, 30 m × 0.250 mm × 0.25 µm film) capillary column. The injector temperature was 250°C, ion source 250°C with electron ionization of 70 eV, the MS scan range m/z 40–400, and helium was used as carrier gas at flow rate of 1 mL/ min with a split ratio of 1:2.

Pyrolysis products were identified with comparison to selected reference compounds with their mass spectra, retention times and by referencing the National Institute of Standards and Technology (NIST) spectral library and literature.[2]

Lignin content by acetyl bromide assay protocol

Acetyl bromide soluble lignin concentration was calculated with the following equation based on the absorbance reading A. In the formula V is the volume of the diluted sample (100 ml), ε is the plant specific extinction coefficient (17.898 g L cm for aspen), m is the weighed mass of the sample (4–6 mg) and L is the path length of the UV beam (1 cm).

Lignin w/w content (%) = 100% * V A / (ε m L)

Field emission scanning electron microscopy (FESEM) protocol

The images were captured using field emission scanning electron microscope (FESEM; Zeiss Sigma VP, Germany) at acceleration voltages of 1.5 to 5 kV using a secondary electron detector and a working distance of 3−5 mm. Prior to imaging, the samples were attached to aluminum SEM stubs with carbon tape followed by sputter-coating using Leica Microsystems EM 600 coating system (Leica Microsystems, Wetzlar, Germany) to deposit a gold - platinum thin layer of 5 nm. Fiji ImageJ software (Research Services Branch, NIH, Bethesda, Maryland, USA) was used for the image analysis.

Gel permeation chromatograms

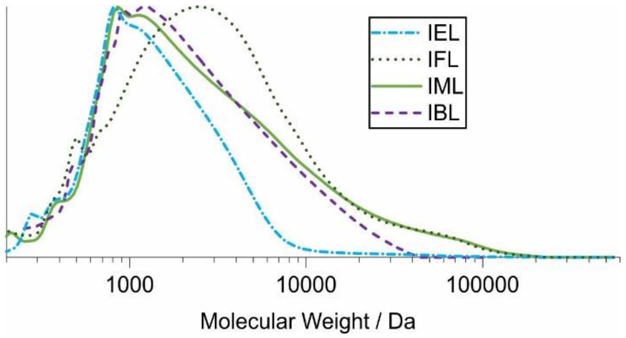

Figure S1. The GPC chromatograms of the acetylated soluble lignin fractions IEL, IFL, IML and IBL in THF.

High performance liquid chromatography (HPLC)

p-hydrobenzoic acid, M = 138

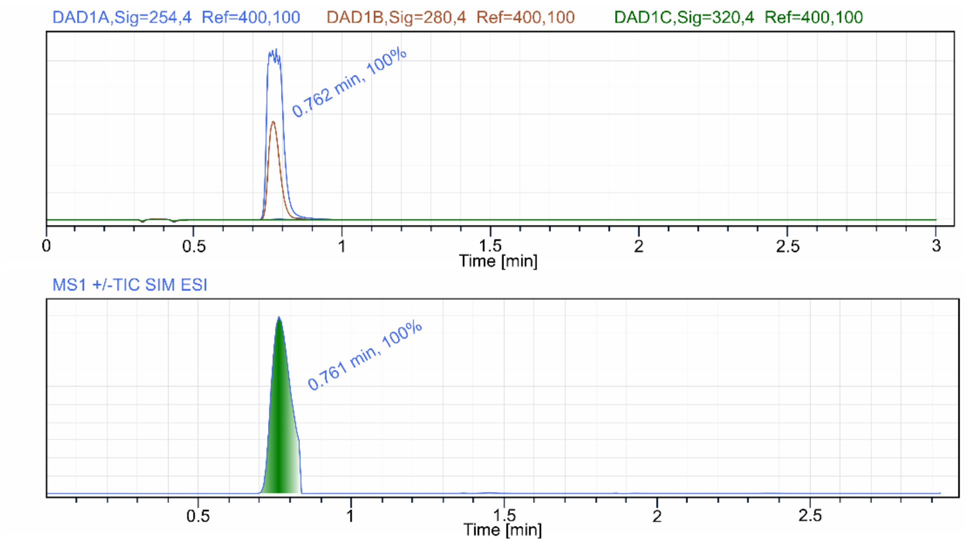

Ethyl acetate extract from 2 M alkali-treated SEL, M = 138

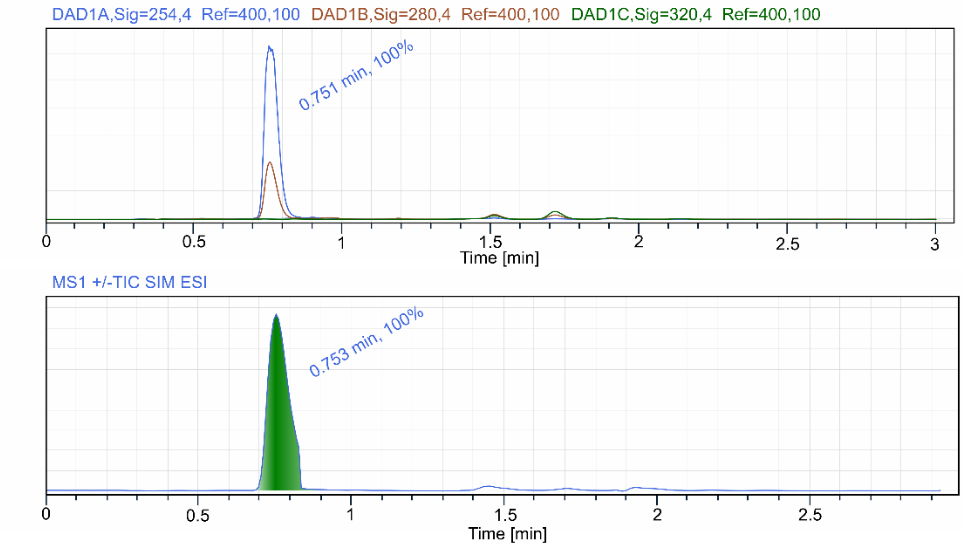

Figure S2. HPLC chromatograms of 2 M NaOH treated SEL, pH-adjusted to 2 and subsequently extracted to ethyl acetate.

Pyrolysis-GC-MS

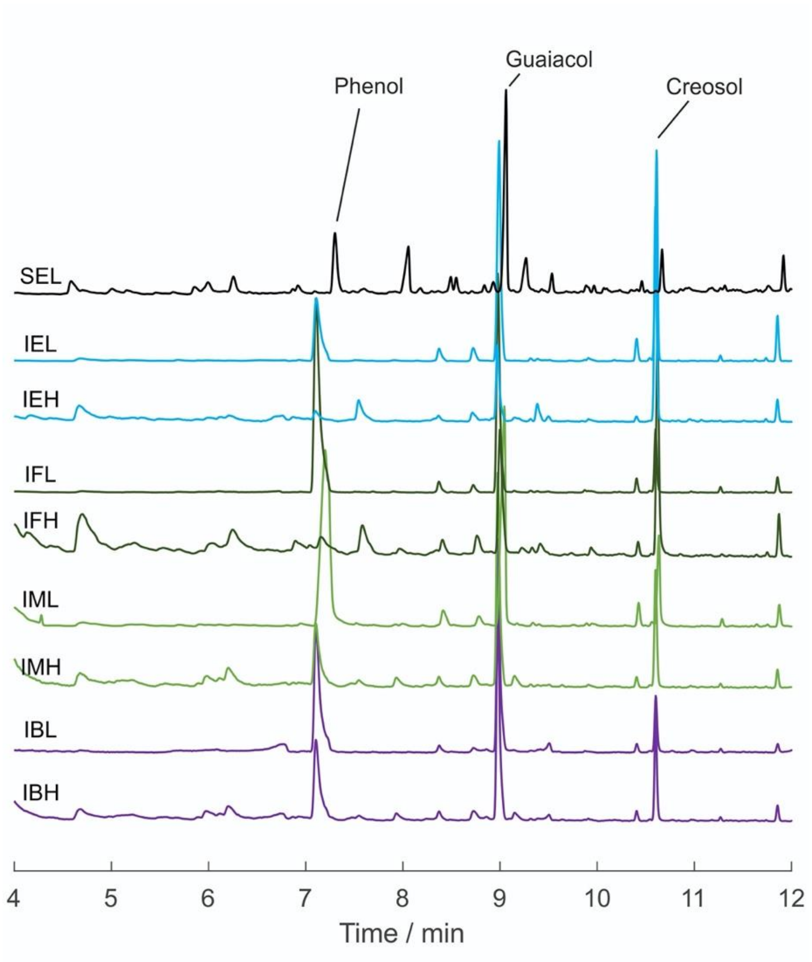

Figure S3. Pyrolysis-GC-MS chromatograms of all soluble and non-soluble fractions in comparison to original SEL at time 4-12 minutes. The chromatograms show that all soluble fractions (IEL, IFL, IML and IBL) contained only minor amounts of carbohydrate originated volatile products. The insoluble fractions on the other hand contained higher amounts of carbohydrate products and either lower (IBH, IEH) or minor (IFH, IMH) amounts of lignin originated volatile compounds.

Nuclear magnetic resonance (NMR) spectral analysis of lignin model compound treated by formic acid

1.)

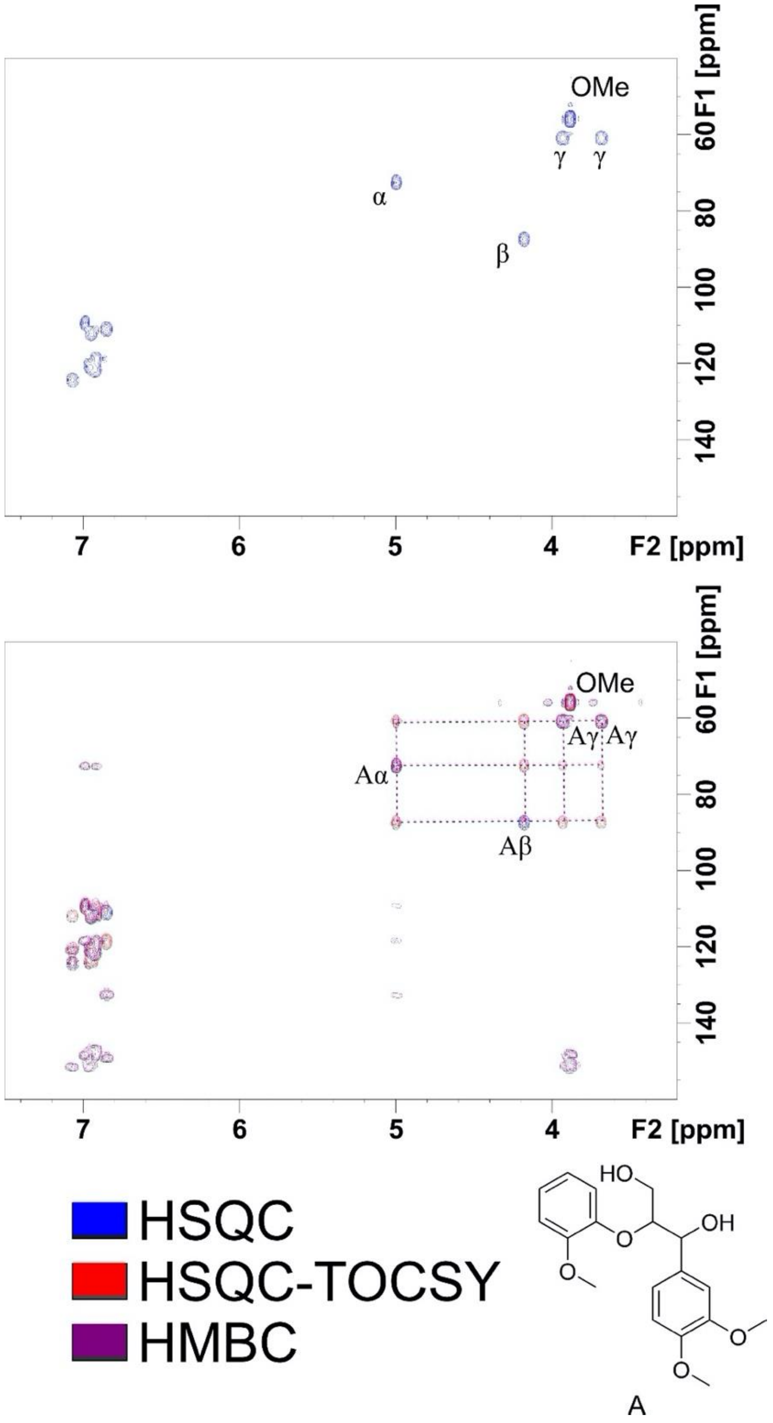

2.)

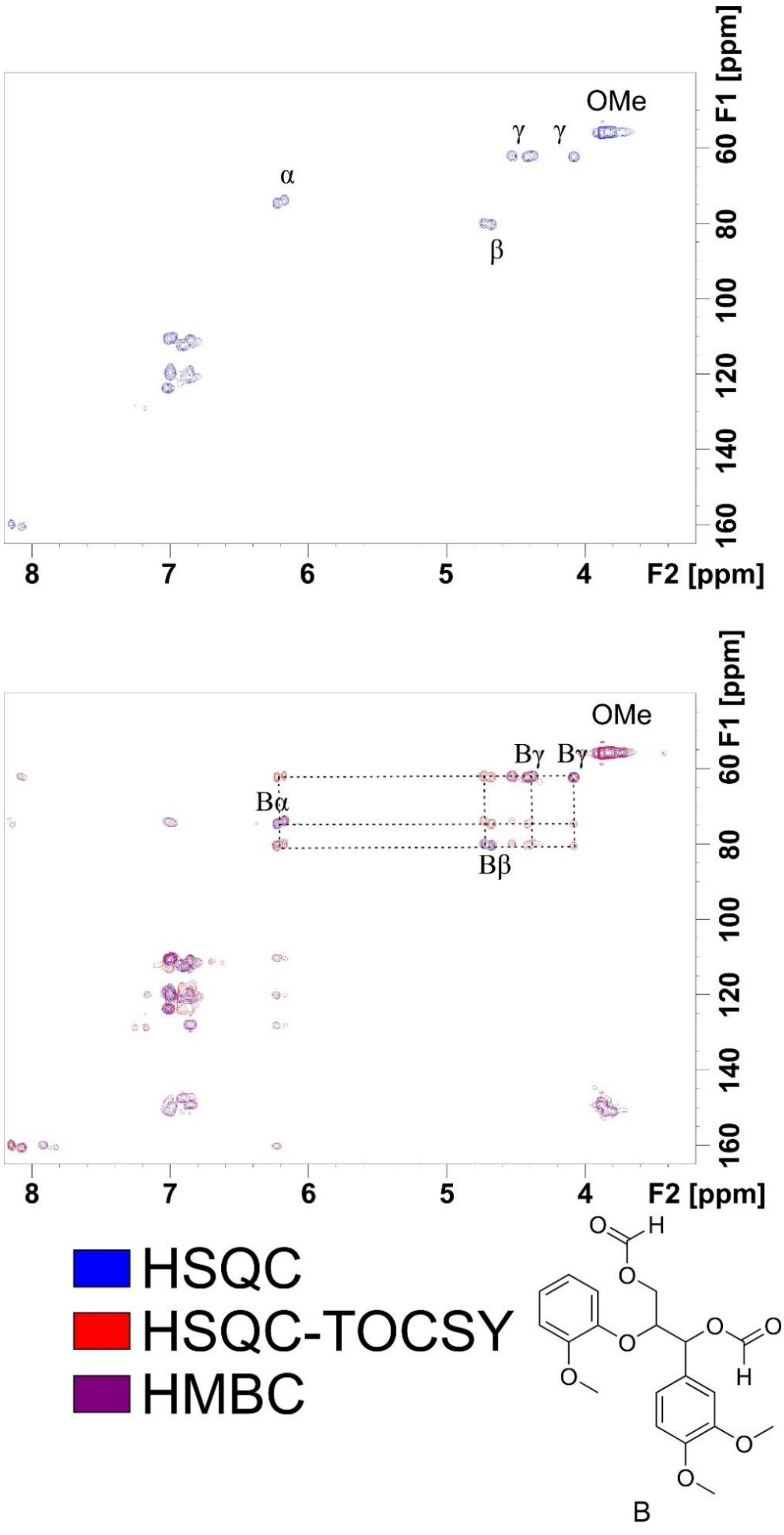

OMe
γ
γ
α
β
60 F1 [ppm]
80
100
120
140
160
8
7
6
5
4
F2 [ppm]
OMe
Bγ
Bγ
Bα
Bβ
HSQC
HSQC-TOCSY
HMBC
B

3.)

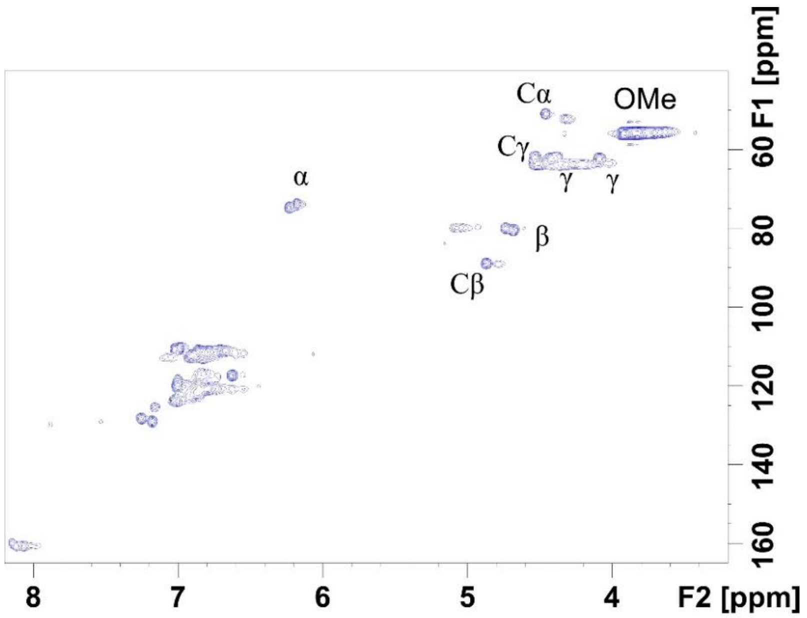
Cα
OMe
Cγ
γ
γ
α
β
Cβ
F1 [ppm]
F2 [ppm]
60
80
100
120
140
160
8
7
6
5
4

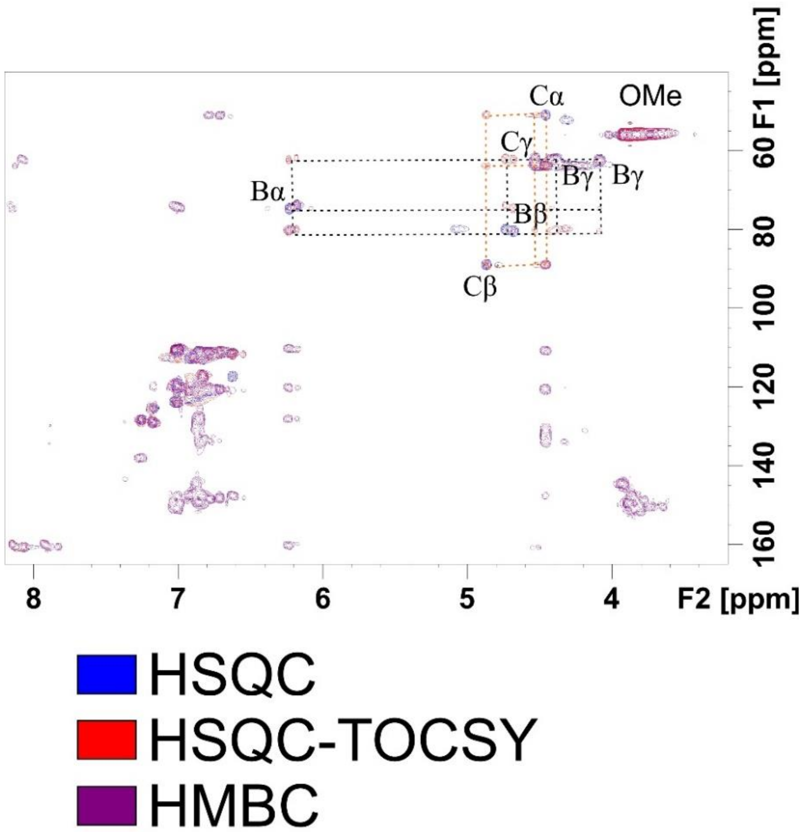
Cα
OMe
Cγ
Bγ
Bγ
Bα
Bβ
Cβ
F1 [ppm]
F2 [ppm]
60
80
100
120
140
160
8
7
6
5
4
HSQC
HSQC-TOCSY
HMBC

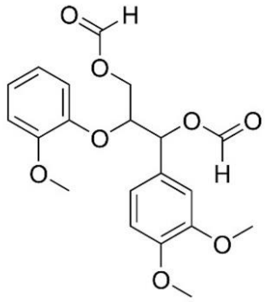

B

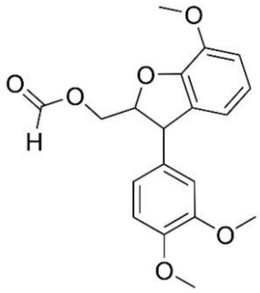

C

4.)

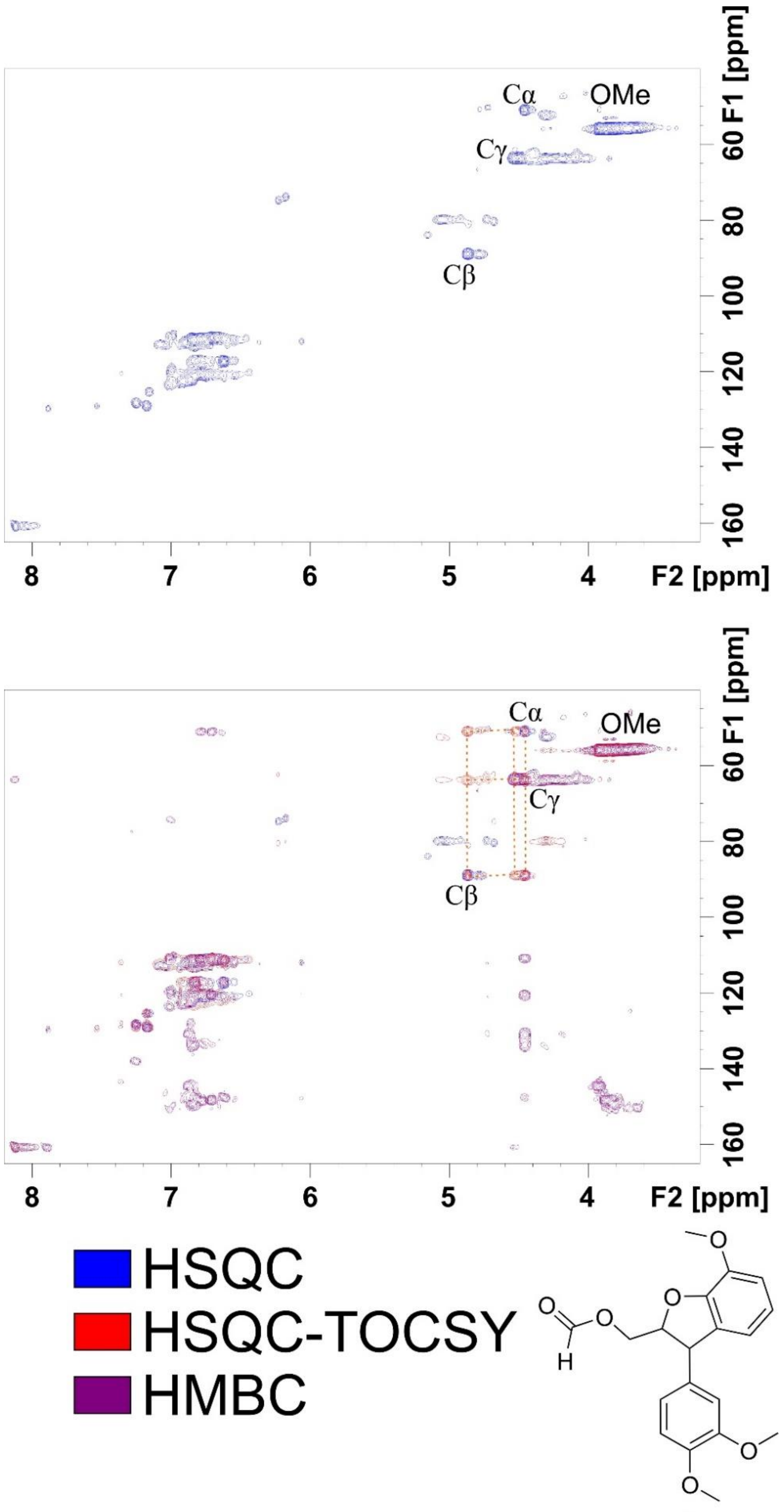

C

5.)

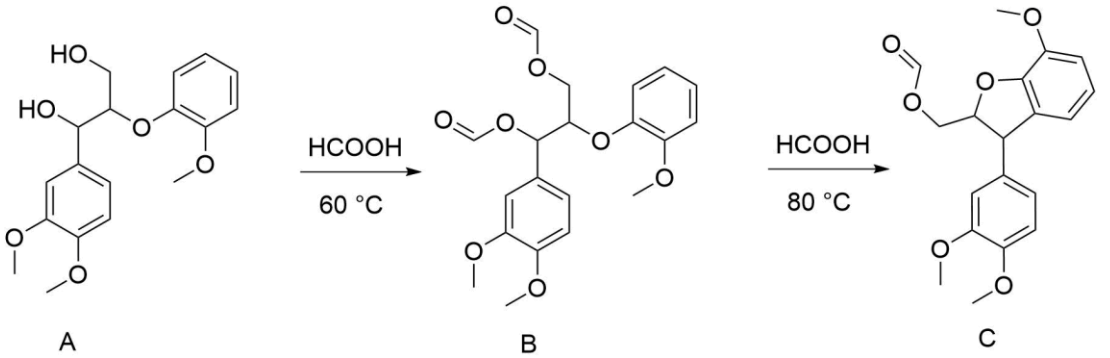

Figure S4. The HSQC, HSQC-TOCSY and HMBC NMR for the formic acid cook of β–O–4 model compound 1-(3,4-Dimethoxyphenyl)-2-(2-methoxyphenoxy)-1,3-propanediol (A) in varying temperatures at 1.) Unreacted starting material (Compound A); 2.) 60°C 0.5 h; 3.) 80°C 0.5 h and 4.) 90°C 0.5 h. The spectra show full esterification (Compound B) at 60°C temperature, followed by formation of cyclic structure (Compound C) at heating up to 90°C. 5.) Reaction pathway in formic acid suggested by NMR data and literature.[3]

HSQC and HMBC of soluble lignin fraction IEL

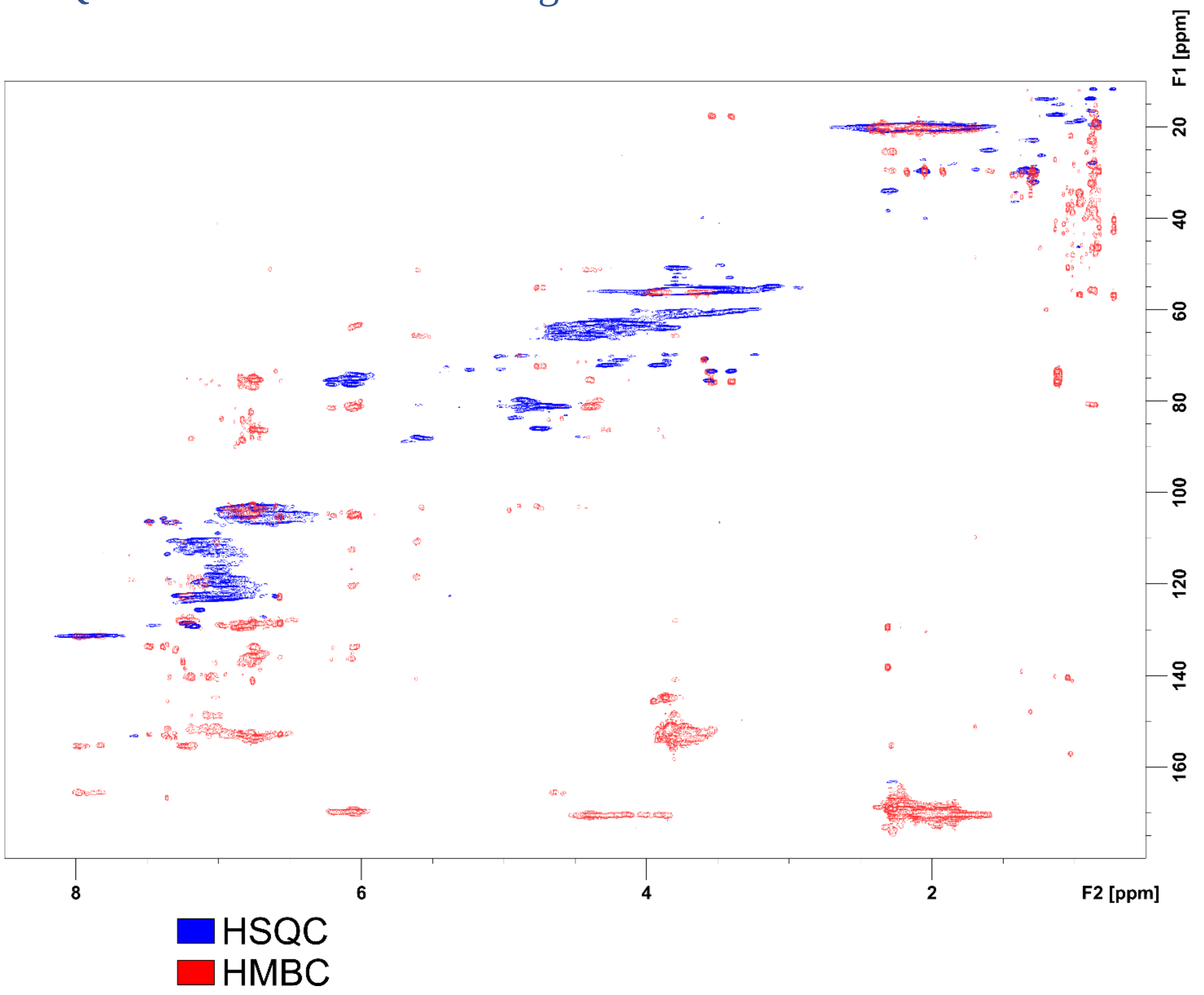

Figure S5. HSQC and HMBC NMR for acetylated IEL fraction shows coupling from the peaks at 3.3-3.5/68-75 ppm to aliphatic region.

^{13}C NMR of the fractionated lignins

A)

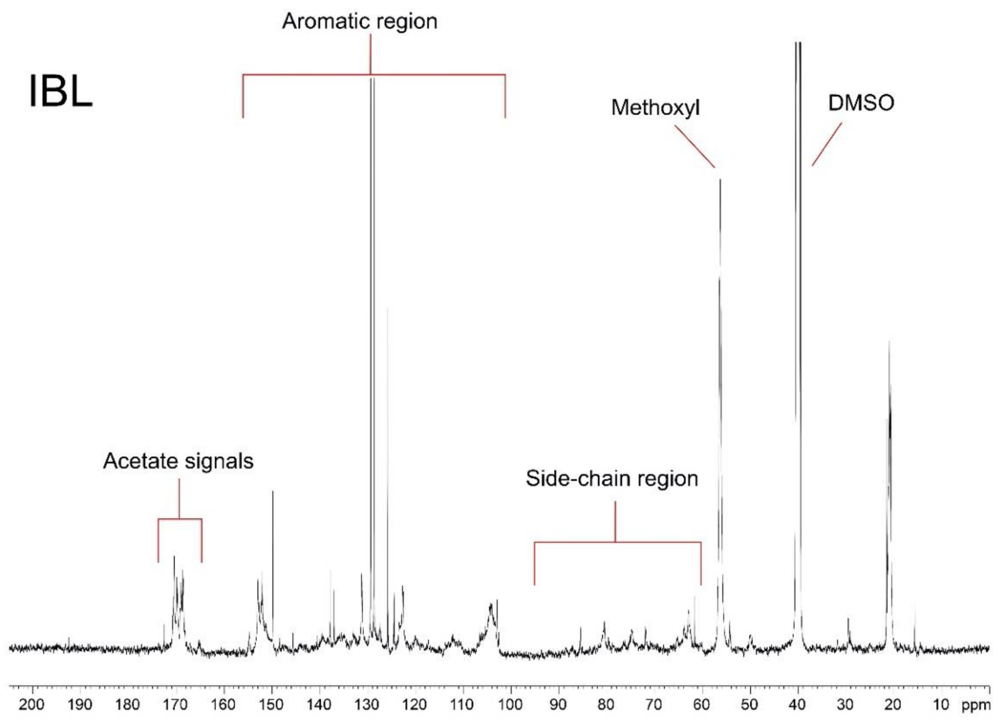

B)

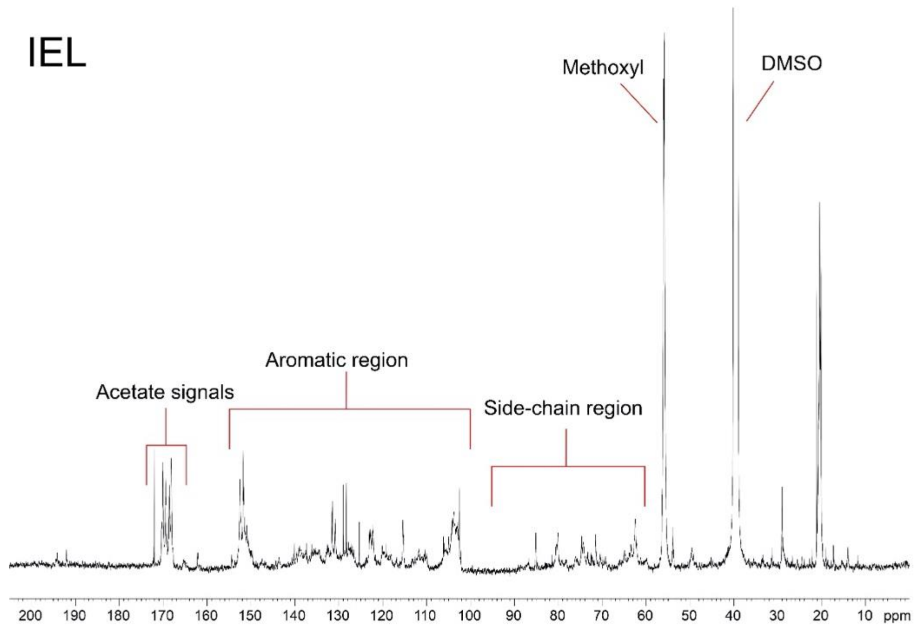

C)

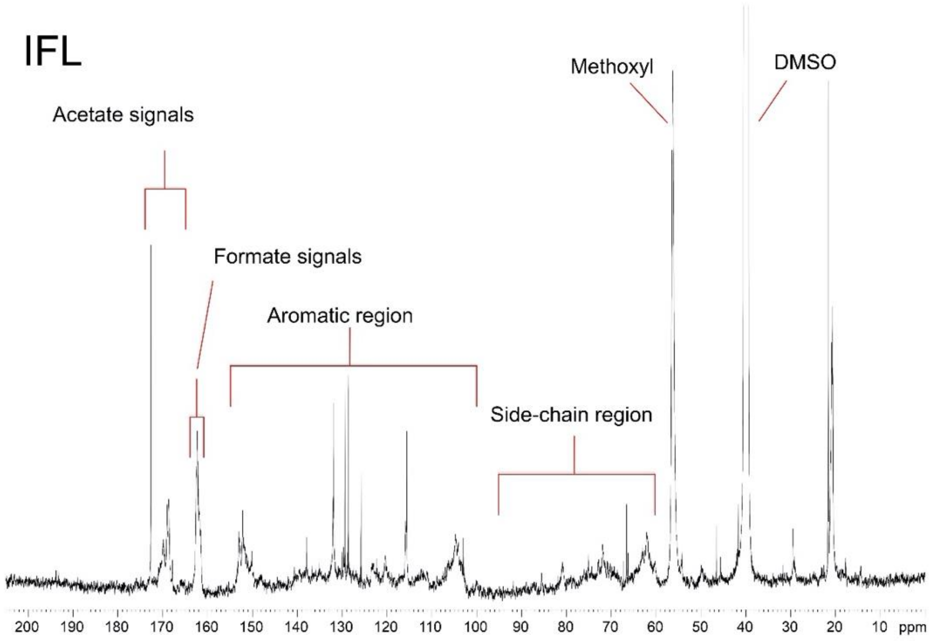

D)

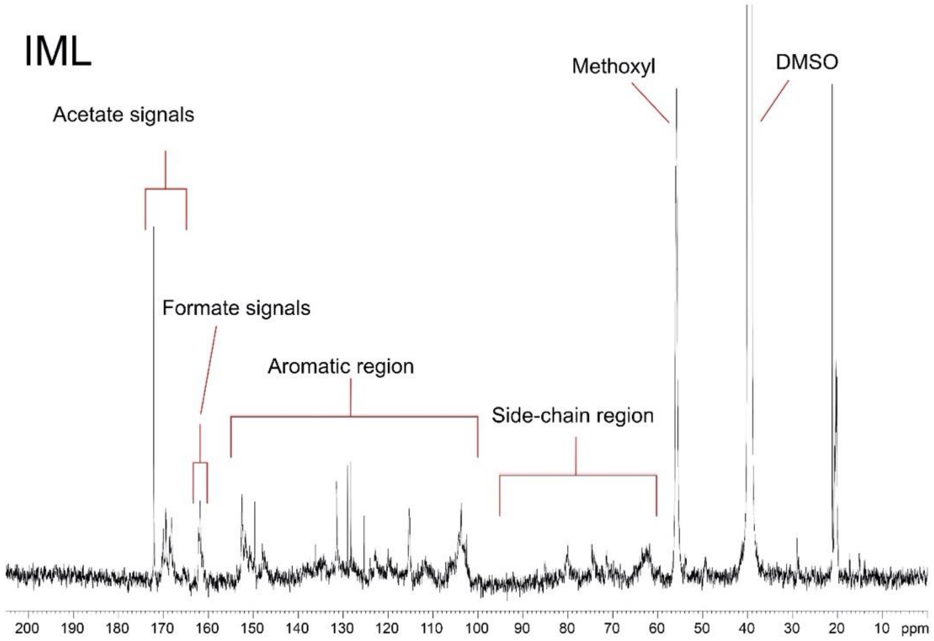

Figure S6. ^{13}C-NMR spectra of soluble fractions. A: IBL, B: IEL, C: IFL and D: IML

^{13}C NMR integrations of Acetate and Formate carbonyl regions

A)

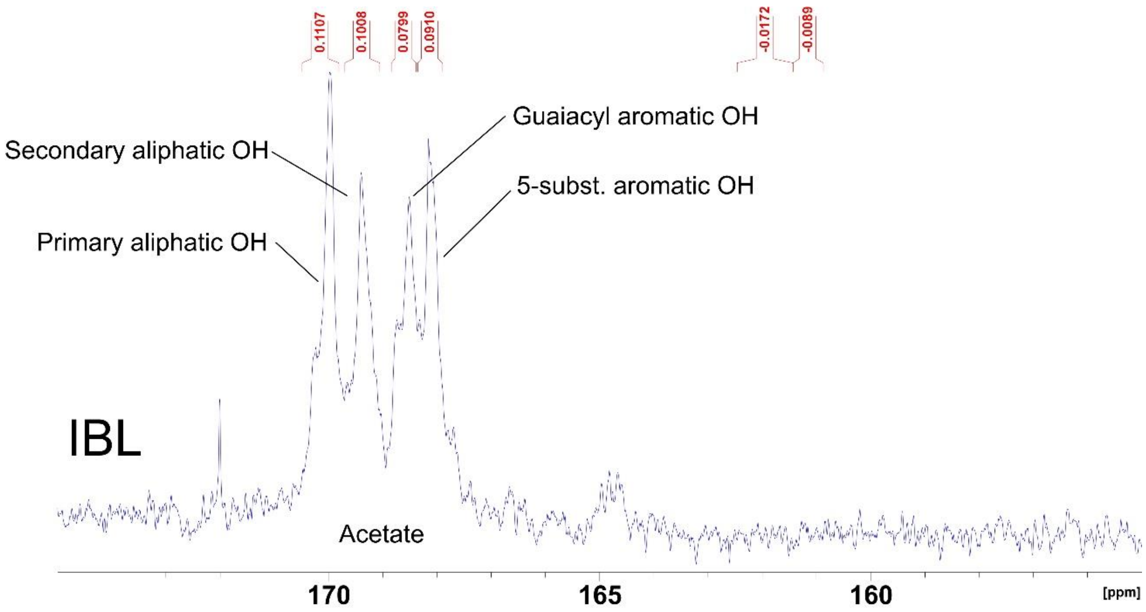

B)

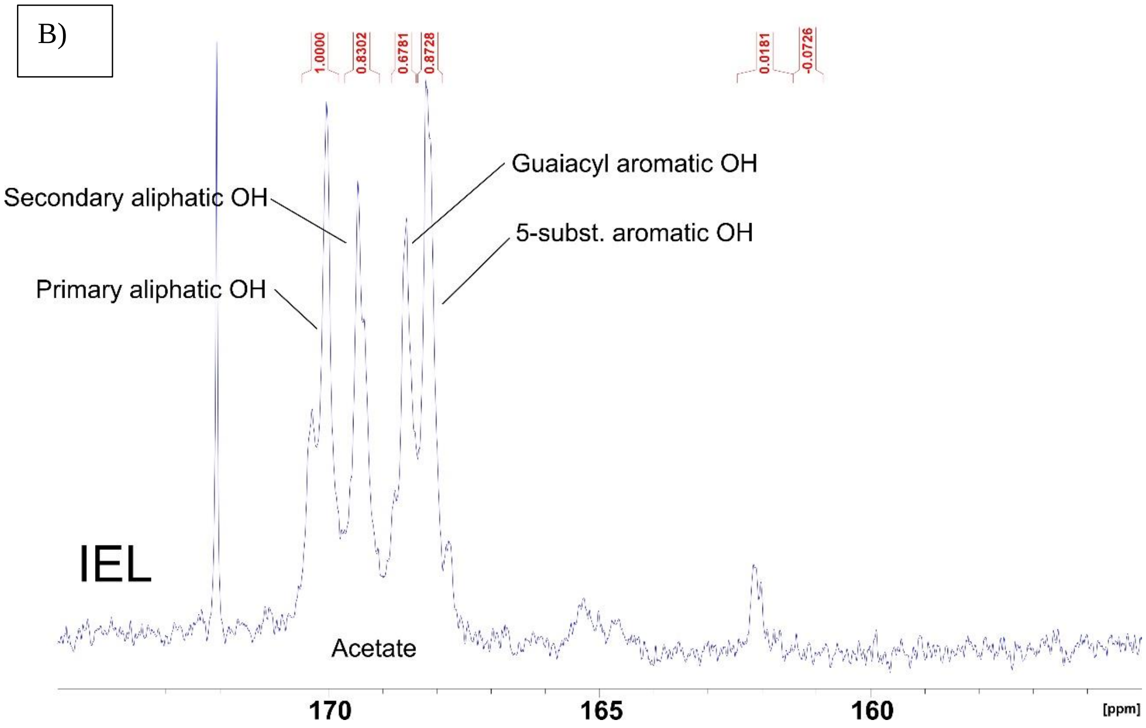

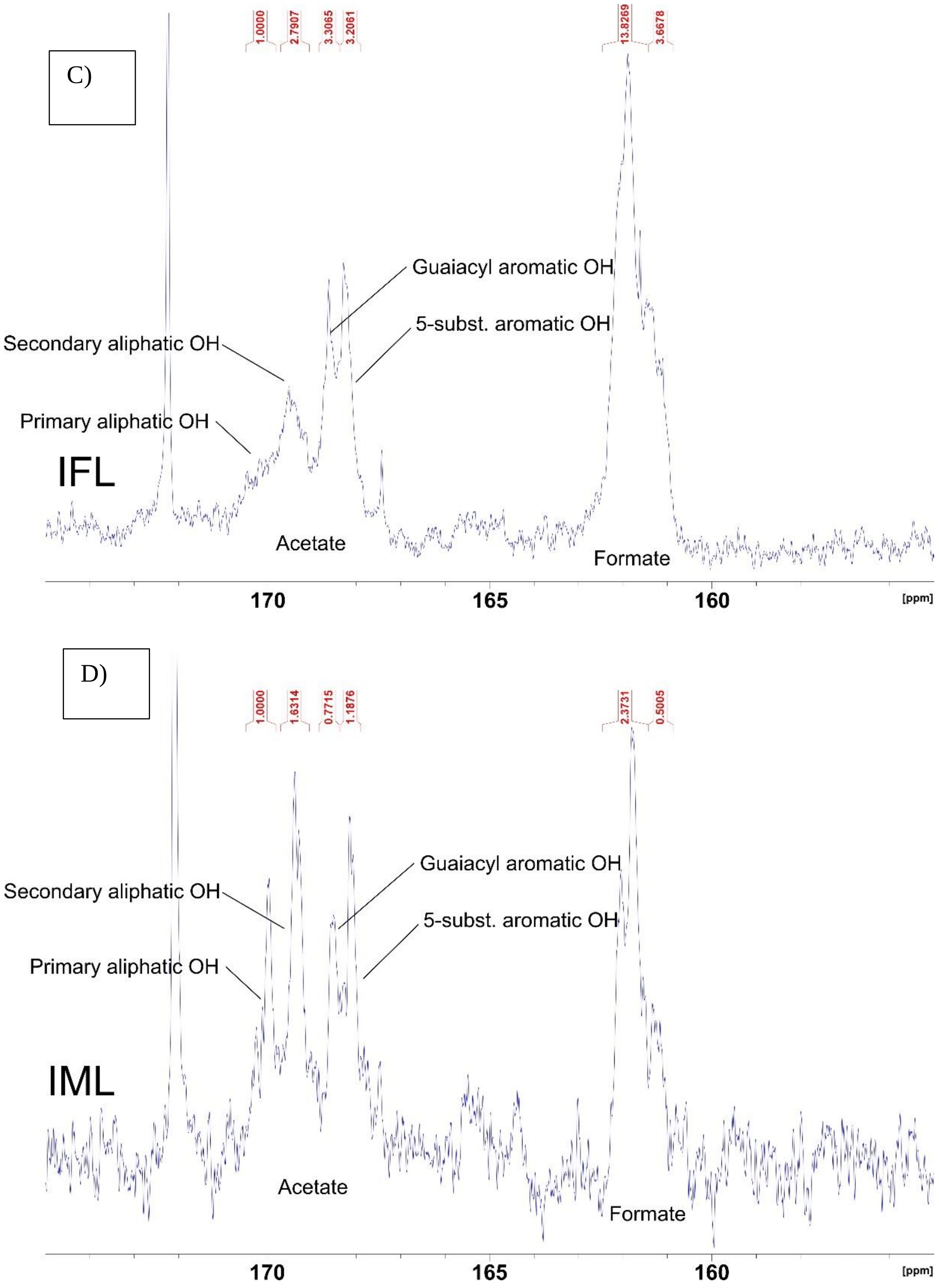

Figure S7. ^{13}C-NMR carbonyl signals of soluble lignin samples A: IBL, B: IEL, C: IFL and D: IML after acetylation. Lignins treated with formic acid (IFL and IML) have both acetate and formate signals after acetylation.

HSQC-NMR integrals of major lignin aromatic groups and side-chain regions

Table S1: HSQC integrated relative volumes of lignin aromatic and side-chain structures. The integrals were calculated relative to 100 S-units in lignin aromatic region and 100 β-O-4 in side-chain region. S= syringyl, S-ox= α-oxidized syringyl, G= guaiacyl, p-BA= para-hydroxybenzoic acid. β-0-4= β-aryl ether, β-5= phenylcoumaran, β-β= resinol.

	IEL	IFL	IML	IBL
S-2,6	100	100	100	100
S-ox-2,6	7.9	7.2	9.1	7.1
G-2,5,6	95.9	97.3	97.5	94.7
p-BA-2,6	18.1	29.4	21.2	22.1
β-0-4	100	100	100	100
β-5	34.6	32.4	38.5	36.3
β-β	26.0	40.2	33.1	27.5

Table S2: HSQC coherences for identified signals in acetylated soluble poplar lignin samples

**HSQC-coherences**	**$^{1}H/^{13}C$ (ppm)**
Hβ-Cβ coupling in phenylcoumaran interunit bonds	3.5/49.1
Hβ-Cβ coupling in resinol interunit bonds	3.1/53.7
H-C coupling in methoxyl groups	3.3–4.0/54.2–57.0
Hγ-Cγ coupling in aryl β-ether interunit bonds	3.7–4.7/63.0
Hγ-Cγ coupling in cinnamyl alcohol end groups	4.7/64.9
Hγ-Cγ coupling in resinol interunit bonds	3.9;4.2/71.4
Hα-Cα coupling in aryl β-ether interunit bonds	5.9/73.9
Hβ-Cβ coupling in aryl β-ether interunit bonds	4.6/79.9
Hβ-Cβ coupling in α-oxidized aryl β-ether interunit bonds	4.6/79.9
Hα-Cα coupling in resinol interunit bonds	4.7/85.1
Hα-Cα coupling in phenylcoumaran interunit bonds	5.6/87.1
H6-C6 and H2-C2 couplings in syringyl groups	6.7/103.2
H6-C6 and H2-C2 couplings in α-oxidized syringyl groups	7.3/106.0
H2-C2 couplings in guaiacyl groups	6.9/111.3
H6-C6 and H5-C5 couplings in guaiacyl groups	6.8/115.2;6.9/119.1
Hα-Cα coupling in cinnamyl alcohol end groups	6.4/130.0
H5-C5 and H3-C3 couplings in *para*-benzoate groups	7.1/122.7
H6-C6 and H2-C2 couplings in *para*-benzoate groups	7.6/131.4

Scheme of lignin formylation and formate ester thermal degradation

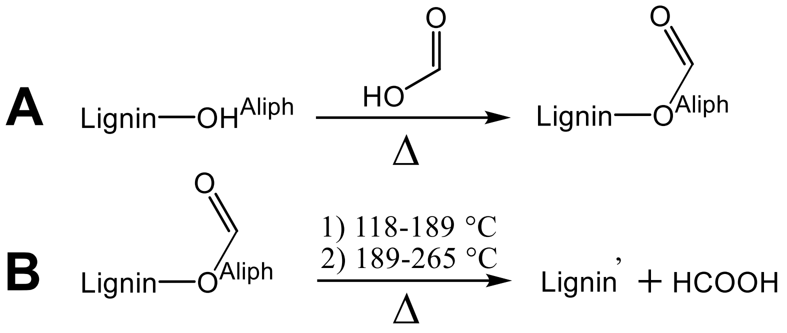

Scheme 1. The proposed reaction pathways for formylation and thermal deformylation of SEL. In (A) aliphatic formate esters are formed both in α- and γ-hydroxyls. In (B) both IFL and IML decompose similarly liberating a formic acid molecule, which further reacts in the dehydration pathway to create carbon monoxide and water or in the decarboxylation pathway to create carbon dioxide and hydrogen.[4,5] Thermal degradation occurs at two stages: First at 118-189°C and second at 189-265°C based on TGA curves.

Scanning electron microscope imaging of the isolated lignins

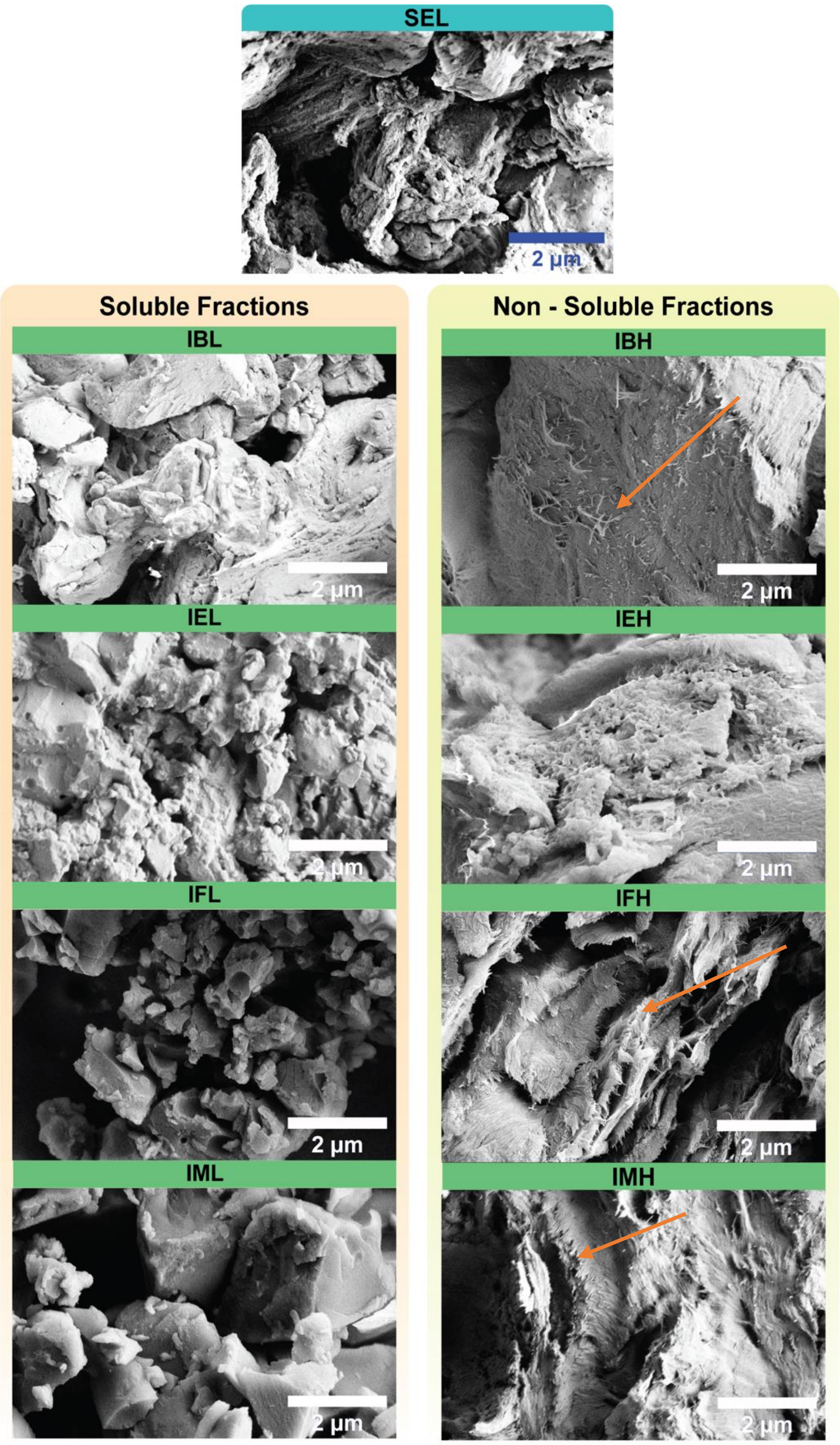

Figure S8. Scanning electron microscopy imaging of the isolated fractions of SEL. Magnification of 15 000 on lignin fractions a) SEL; b) IBL; c) IBH; d) IEL; e) IEH; f) IFL; g) IFH; h) IML; i) IMH. The scale bar in each is 2 µm. The orange arrows show cellulose fibers.

References

(1) Nakatsubo, F.; Sato, K.; Higuchi, T. Synthesis of Guaiacylglycerol-β-Guaiacyl Ether. *Holzforschung* **1975**, *29* (5), 165–168. https://doi.org/10.1515/hfsg.1975.29.5.165.

(2) Kuuskeri, J.; Häkkinen, M.; Laine, P.; Smolander, O.-P.; Tamene, F.; Miettinen, S.; Nousiainen, P.; Kemell, M.; Auvinen, P.; Lundell, T. Time-Scale Dynamics of Proteome and Transcriptome of the White-Rot Fungus Phlebia Radiata: Growth on Spruce Wood and Decay Effect on Lignocellulose. *Biotechnol Biofuels* **2016**, *9* (1), 192. https://doi.org/10.1186/s13068-016-0608-9.

(3) Ede, R.; Brunow, G.; Poppius, K.; Sundquist, J.; Hortling, B. Formic Acid/Peroxyformic Acid Pulping. *Nord Pulp Paper Res J* **1988**, *3* (3), 119–123. https://doi.org/10.3183/npprj-1988-03-03-p119-124.

(4) Moriyoshi, T.; Sam, K.; Uosaki, Y. Hydrothermal Decomposition of Esters under High Pressure. *High Press Res* **2001**, *20* (1–6), 491–505. https://doi.org/10.1080/08957950108206197.

(5) Ren, W.; Spearrin, M. R.; Davidson, D. F.; Hanson, R. K. Experimental and Modeling Study of the Thermal Decomposition of C3–C5 Ethyl Esters Behind Reflected Shock Waves. *J Phys Chem A* **2014**, *118* (10), 1785–1798. https://doi.org/10.1021/jp411766b.
